# Saturated very long-chain fatty acids regulate macrophage plasticity and invasiveness

**DOI:** 10.1186/s12974-022-02664-y

**Published:** 2022-12-17

**Authors:** Bettina Zierfuss, Agnieszka Buda, Andrea Villoria-González, Maxime Logist, Jure Fabjan, Patricia Parzer, Claire Battin, Streggi Vandersteene, Inge M. E. Dijkstra, Petra Waidhofer-Söllner, Katharina Grabmeier-Pfistershammer, Peter Steinberger, Stephan Kemp, Sonja Forss-Petter, Johannes Berger, Isabelle Weinhofer

**Affiliations:** 1 Department of Pathobiology of the Nervous System, Center for Brain Research, Medical University of Vienna, Spitalgasse 4, 1090 Vienna, Austria; 2 Department of Neuroscience, Centre de Recherche du CHUM, Université de Montréal, Montréal, H2X 0A9 Canada; 3 Department of Chronic Diseases and Metabolism, Translational Research in GastroIntestinal Disorders, KU Leuven, 3000 Leuven, Belgium; 4 Division of Immune Receptors and T Cell Activation, Institute of Immunology, Center for Pathophysiology, Infectiology and Immunology, Medical University of Vienna, 1090 Vienna, Austria; 5 Genetic Metabolic Diseases, Department of Clinical Chemistry, Amsterdam University Medical Center, Amsterdam Neuroscience, Amsterdam Gastroenterology Endocrinology Metabolism, University of Amsterdam, 1105 AZ Amsterdam, The Netherlands

**Keywords:** Extracellular matrix degradation, Immune response, Lipid metabolism, Neuroinflammation, X-linked adrenoleukodystrophy

## Abstract

Saturated very long-chain fatty acids (VLCFA, ≥ C22), enriched in brain myelin and innate immune cells, accumulate in X-linked adrenoleukodystrophy (X-ALD) due to inherited dysfunction of the peroxisomal VLCFA transporter ABCD1. In its severest form, X-ALD causes cerebral myelin destruction with infiltration of pro-inflammatory skewed monocytes/macrophages. How VLCFA levels relate to macrophage activation is unclear. Here, whole transcriptome sequencing of X-ALD macrophages indicated that VLCFAs prime human macrophage membranes for inflammation and increased expression of factors involved in chemotaxis and invasion. When added externally to mimic lipid release in demyelinating X-ALD lesions, VLCFAs did not activate toll-like receptors in primary macrophages. In contrast, VLCFAs provoked pro-inflammatory responses through scavenger receptor CD36-mediated uptake, cumulating in JNK signalling and expression of matrix-degrading enzymes and chemokine release. Following pro-inflammatory LPS activation, VLCFA levels increased also in healthy macrophages. With the onset of the resolution, VLCFAs were rapidly cleared in control macrophages by increased peroxisomal VLCFA degradation through liver-X-receptor mediated upregulation of ABCD1. ABCD1 deficiency impaired VLCFA homeostasis and prolonged pro-inflammatory gene expression upon LPS treatment. Our study uncovers a pivotal role for ABCD1, a protein linked to neuroinflammation, and associated peroxisomal VLCFA degradation in regulating macrophage plasticity.

**Supplementary Information:**

The online version contains supplementary material available at 10.1186/s12974-022-02664-y.

## Background

Tight regulation of lipid metabolism is essential for the innate immune response. This is especially evident in macrophages, for which reprogramming the lipid composition in response to different activation signals is key for their opposing roles in pro- and anti-inflammatory processes [1]. The metabolic program that supports pro-inflammatory macrophage reactions relies on both, glycolysis to cover the rapid energy demand and lipogenesis to produce pro-inflammatory mediators and saturated fatty acids for membrane reconfiguration [2, 3].

Recently, an unexpected role for extracellular saturated very long-chain fatty acids (VLCFAs, ≥ C22) in modulating pro-inflammatory signalling in human monocytes as well as murine bone marrow-derived macrophages was identified [4]. In their study, Kanoh et al. demonstrated that serum-derived GM3 gangliosides enriched with saturated VLCFAs enhanced LPS-induced signalling of the pattern recognition receptor toll-like receptor 4 (TLR4), whereas gangliosides containing saturated long-chain fatty acids (LCFAs) such as C16:0 had an antagonistic effect [4]. Synthesis of saturated VLCFAs is accomplished by a family of ER-embedded substrate-specific enzymes called elongation of very long-chain fatty acids (ELOVL) proteins 1–7 that catalyse the first, rate-limiting step within the LCFA and VLCFA elongation cycle. Degradation of VLCFAs is localized in peroxisomes, where the fatty acids are broken down by β-oxidation. Under physiological conditions, VLCFAs are of low abundance but they accumulate strongly in tissues and body fluids of patients affected by the inherited neurodegenerative disorder X-linked adrenoleukodystrophy (X-ALD, OMIM #300100). In X-ALD, mutations in the *ATP-binding* *cassette* *subfamily* *D* *member* *1* (*ABCD1*) gene inactivates the function of the encoded VLCFA transporter, resulting in impaired degradation of VLCFAs within peroxisomes [5, 6]. In the severest neuroinflammatory presentation (cerebral X-ALD, CALD), patients suffer from rapidly progressive myelin destruction and axonal damage with infiltration of peripheral immune cells such as monocytes and T cells [5, 7]. If the inflammatory demyelination in CALD is detected at an early stage, the degradation of myelin and axons can be stopped by haematopoietic stem cell (HSC) transplantation or gene therapy [8–10]. Of note, the HSC-derived immune cell lineage most affected by VLCFA accumulation in X-ALD are monocytes/macrophages [11]. Thus, these cells are not only central in the onset and progression of CALD but, upon correction by HSC-transplantation, can also stop the neuroinflammation in affected patients. Consistent with this concept, we previously demonstrated that X-ALD macrophages are pro-inflammatory skewed and that their plasticity to adapt an anti-inflammatory phenotype is impaired [12]. Exactly how VLCFA levels contribute to the pro-inflammatory activation state of macrophages is unclear.

In this study, we specifically investigated the role of saturated VLCFAs in macrophage activation. We found that excessive intracellular VLCFAs prime the macrophage membrane for pro-inflammatory responses and, when added extrinsically to macrophages, activate the c-Jun N-terminal kinase (JNK) pathway cumulating in the release of pro-inflammatory chemokines. Following an acute pro-inflammatory response, normal macrophages rapidly cleared saturated VLCFAs by increased peroxisomal β-oxidation involving LXR-mediated upregulation of the VLCFA transporter ABCD1. Consequently, in X-ALD macrophages, the ABCD1 deficiency and impaired ability to degrade saturated VLCFAs prolonged the pro-inflammatory gene expression pattern. Accordingly, our data uncover a pivotal role for ABCD1 and the associated peroxisomal β-oxidation of VLCFAs in resolving the inflammatory state of macrophages.

## Materials and methods

### X-ALD patients and healthy volunteers

The study included peripheral blood samples from 11 adult X-ALD patients (age 23–45 years, mean = 36 years) and 15 healthy volunteers (age 24–61 years, mean = 40 years). X-ALD patients displayed clinical symptoms of axonopathy in the spinal cord but no signs of cerebral involvement (CALD) at MRI. The study was approved by the Ethical Committee of the Medical University of Vienna (EK1462/2014) and informed consent was obtained from participating X-ALD patients and healthy volunteers. In addition, leukoreduction system chambers derived from 46 healthy donors (median age = 33) were purchased from the General Hospital of Vienna, Austria, and used for monocyte isolation.

### Isolation of human monocytes from peripheral blood

Primary CD14+ monocytes were isolated from the peripheral blood by Ficoll density-gradient centrifugation (PAN-Biotech) and positive selection for CD14+ cells using MACS microbeads and the LS column system (Miltenyi Biotec) according to the manufacturer’s instructions.

### Differentiation and activation of human macrophages

CD14+ monocytes were differentiated in RPMI medium (Sigma Aldrich) containing 1% penicillin/streptomycin, 1% glutamine, 1% Fungizone (all Invitrogen) and 10% FCS (Gibco Life Technologies), supplemented with 50 ng/ml human recombinant M-CSF (PeproTech) for 7 days. After differentiation, either the LXR agonist T0901317 (Calbiochem), the LXR antagonist GSK1440233 (GlaxoSmithKline, [13]) or the natural LXR ligand 25-hydroxycholesterol (25-HC, Merck) was added for up to 24 h. For time response analysis, M-CSF-differentiated macrophages were stimulated with 100 ng/ml LPS (*Escherichia* *coli* 055:B5, Cat.no. L4005, Sigma) or 100 µM C26:0 (Merck, dissolved in EtOH) as indicated. C16:0 (Merck) was dissolved in EtOH and used at a final concentration of 100 µM.

### Jurkat and THP-1 reporter assays

Jurkat reporter cells expressing E6-1-NFκB::eGFP-TLR2/1, E6-1-NFκB::eGFP-TLR2/6 or E6-1-NFκB::eGFP-TLR4/CD14, as well as reporter THP-1 E6-1-NFκB::eGFP-TLR4/CD14 cells were previously described [14, 15]. Cells (5 × 10/well) were stimulated with TLR ligands flagellin, LPS, PAM3CSK4 or MALP2 (all purchased from Invivogen, San Diego, CA and used at a final concentration of 100 ng/ml) or saturated LCFA (C16:0), saturated VLCFAs (C24:0, C26:0) or mono-unsaturated LCFA (C18:1) in 96 well plates. After 24 h, cells were harvested and NFκB-eGFP expression was assessed via flow cytometry using FACS Calibur with CellQuest software (both BD Biosciences, San Jose, CA). Data were analysed using FlowJo software version 10.6.1 (Tree Star, Ashland, OR).

### RNA isolation and RT-qPCR

RNA was isolated from differentiated macrophages using the RNeasy Mini Kit (Qiagen) according to the manufacturer’s instructions. cDNA was synthesized from total RNA samples using the iScript™ cDNA synthesis Kit (Bio-Rad). qPCR was performed with the CFX96™ Real-Time PCR Detection System (Bio-Rad) for each cDNA sample in technical duplicates. Relative mRNA levels were detected by SYBRGreen incorporation and calculated by the 2^−∆∆Cq^ method using *HPRT1* or *RACK1* for normalization and a control sample on each plate as internal reference. To determine the absolute mRNA abundance of *ABCD1*, and for normalization, *HPRT1*, quantification was carried out using standard curves of known copy numbers of linearized plasmids containing ABCD1 and HPRT1 cDNA. Sequences of primers are listed in Additional file 1: Table S1.

### RNA-seq and bioinformatic analysis of the data

First, the quality of RNA was assessed by using the Agilent 2100 Bioanalyzer (Agilent Technologies). All used samples had RNA integrity number (RIN) values higher than 8.6. Next, mRNA was selected using poly-A enrichment, followed by the cDNA library construction. The sequencing was performed at the Biomedical Sequencing Facility of the Medical University of Vienna, Austria, on Illumina HiSeq2000 using single end 50 bp reads and 10 samples per lane. The initial mapping of reads to the hg38 human genome assembly was done using STAR [16]. Count tables were produced and further analysis was performed with R 4.0.0 using DESeq2 [17]. The likelihood ratio test was performed to test the effect of the group, with the reduced model containing the isolation time only. The comparison (cells from X-ALD patients to cells from healthy controls) was performed with alpha set to 0.01 using the Benjamini–Hochberg method for adjusting *p*-values. The data were corrected for gene length and then tested using the Wallenius approximation. The resulting *p*-values were corrected using the Benjamini–Hochberg method. The results from RNAseq analysis were further interpreted using the following workflows: (1) GO Biological Processes (GO:BP) annotation enrichment analysis was performed using Enrichr (https://maayanlab.cloud/Enrichr/) as previously described [18, 19]; (2) biological network analysis and visualization was performed in NetworkAnalyst 3.0 (https://www.networkanalyst.ca/) and inbuilt generic PPI database (IMEx Interactome), with a minimal network being used as described [20]; and (3) volcano plot of differentially expressed genes belonging to GO:BP was prepared using R 4.0.0 with ggplot2, ggrepel and ggsci.

### Western blot analysis

Differentiated macrophages were lysed in RIPA buffer containing protease inhibitors (Roche cOmplete), mixed with 5× sample buffer before separation of proteins on a denaturing 7.5% polyacrylamide gel by discontinuous electrophoresis (SDS–PAGE), followed by semidry blotting onto nitrocellulose membrane. To determine the phosphorylation states of proteins, samples were treated with a cocktail of protease and phosphatase inhibitors (1× Roche cOmplete protease inhibitors, 1× PhosStop (Sigma), 1 mM Na_3_VO_4_, 1 mM NaF, 1 mM PMSF). After the blot-transfer, equal protein load was monitored by Ponceau staining. All blots were blocked with 4% non-fat dry milk powder (w/vol) in TBS-T and probed with primary antibodies against the human ABCD1 protein (Euromedex ALD-1D6-AS, clone 2AL-1D6, 1:10,000), rabbit anti-human SAPK/JNK (Cell Signaling, #9252; 1:1000), rabbit anti-human Phospho-SAPK/JNK (Thr183/Tyr185; Cell Signaling, #9251; 1:1000), rabbit anti-human NF-κB p65 (Cell Signaling Technology, #4764, 1:1000), rabbit anti-human phospho-NF-κB p65 (Ser536; Cell Signaling Technology, #3033, 1:1000) and mouse anti-human β-actin (Chemicon, 1:100,000) followed by goat anti-mouse or anti-rabbit secondary antibodies conjugated with horseradish peroxidase (Dako, 1:30,000). Blots probed with anti-phosphorylated NF-κB p65 and anti-phosphorylated JNK antibody, were stripped with acidic stripping buffer (0.2 M glycine, 0.5 M NaCl, pH 2.5) three times for 5 min and washed thoroughly with TBS-T before re-probing with anti-NF-κB p65 or anti-JNK to enable normalization to protein levels. For detection, the Immobilon Western HRP Substrate Peroxide Solution and Immobilon Reagent (Millipore) were used with the ChemiDoc Imaging System and Image Lab software (Bio-Rad).

### Cytokine measurement

The chemokines CXCL8, CCL3, CCL4, CCL11, CX3CL1, CXCL1, CCL7, IL-12p40, CCL22, IL-15, CXCL10 and CCL2 were measured in the supernatant collected from cultured macrophages using Luminex® bead array technology (HCYTOMAG-60K-06.Hum, Merck) according to the manufacturer’s instruction.

### Fatty acid analysis

The total amounts of saturated, mono-unsaturated and polyunsaturated fatty acids were determined by electrospray ionization mass spectrometry (ESI–MS) as described by Valianpour et al. [21] and normalized to macrophage protein content.

### β-Oxidation of ^14^C-labelled C16:0 and C26:0

Radiolabelled fatty acids, [1-^14^C]-palmitic acid (C16:0; ARC 0172A) and [1-^14^C]-hexacosanoic acid (C26:0; ARC 1253), were obtained from American Radiolabeled Chemicals (St. Louis, MO, USA). Free fatty acids in ethanol were aliquoted into glass reaction tubes, dried under a stream of nitrogen and solubilized in 10 mg/ml α-cyclodextrin by ultrasonication. The reaction mix contained 4 µM of labelled fatty acids, 2 mg/ml α-cyclodextrin, 30 mM KCl, 8.5 mM ATP, 8.5 mM MgCl_2_, 1 mM NAD^+^, 0.17 mM FAD, 2.5 mM l-carnitine, 0.16 mM CoA, 0.5 mM malate, 0.2 mM EDTA, 1 mM DTT, 250 mM sucrose and 20 mM Tris–Cl, pH 8.0. Reactions were started by addition of 5 × 10^6^–2 × 10^7^ cells, carried out for 1 h at 37 °C and stopped by addition of KOH and heating to 60 °C for 1 h. After protein precipitation by HClO_4_, a Folch partition was carried out, and ^14^C-acetate was determined in the aqueous phase by scintillation counting using the Perkin Elmer Tri-Carb 4910TR Scintillation Counter.

### Flow cytometry analysis

For detachment, adherent macrophages were washed with PBS, incubated with 300 µl 10× Gibco™TrypLE™Select (Gibco, Life Technologies) at 37 °C for 15 min and gently collected with a cell scraper after adding 300 µl PBS. Before staining, Fcγ receptor blockage was performed by incubating the cells with 3 mg/ml Beriglobin P (#I4506, Sigma Aldrich) at 4 °C in the dark for 30 min. To determine the purity of isolated primary CD14+ monocytes, cells were stained with either FITC-conjugated mouse anti-human CD14+ REAfinity (#130-110-518, Miltenyi Biotec), FITC-conjugated human IgG1 REAfinity control (#130-113-437, Miltenyi Biotec), PE-conjugated mouse anti-human CD19 monoclonal (clone LT19, #130-091-247, Miltenyi Biotec), PE-conjugated monoclonal isotype control (#120-002-723, Miltenyi Biotec), FITC-conjugated mouse anti-human CD3 monoclonal (#130-080-401, Miltenyi Biotec) and FITC-conjugated mouse IgG2a isotype control (#130-091-837, Miltenyi Biotec) antibodies. To determine the frequency of CD86+ macrophages, cells were stained using anti-CD86-PerCP-eFluor 710 (eBioscience) or isotype control (IgG2b-PerCP-eFluor-710, eBioscience) antibodies. Data were acquired using a BD LSRFortessa™ flow cytometer and analysed using FlowJo software, Treestar Inc.

### Calcein Red-AM viability assay

The viability of human primary macrophages exposed to VLCFAs was investigated using Calcein Red-AM staining. Cells were incubated with C26:0 (10, 20, 50 or 100 µM with final concentration of EtOH filled up to 1%) for 24 h, washed with PBS and stained for 30 min with 1 µM Calcein Red-AM (Biolegend). Plates were imaged using the IncuCyte SX5 live-cell analysis system (Sartorious) with a 10× objective for phase contrast and the orange channel (acquisition time 400 ms). Images were examined using the IncuCyte software. Phase images were subjected to segmentation adjustment of + 1. The analysis definition was optimized for the red channel, so that each red object corresponded to a viable cell by performing a Top-Hat segmentation with a threshold of 15 OCU, a radius of 10 µm, and an edge sensitivity of − 25. By using these settings, red object areas smaller than 80 µm^2^ were filtered out. Data were represented as the number of viable cells per image against phase area per image.

### Immunofluorescence

Macrophages were in vitro differentiated for 7 days on poly-l lysine coated coverslips before addition of C26:0 (100 µM) or the solvent ethanol. After 24 h of treatment, cells were washed with PBS, fixed in 3% paraformaldehyde for 20 min and again washed with PBS. For immunofluorescence, cells were permeabilized with 0.1% Triton for 5 min and blocked with 2% FCS, 2% BSA and 0.2% fish gelatin for 30 min, followed by PBS washing. For vinculin staining, cells were incubated for 2 h with a monoclonal mouse anti-human vinculin antibody (1:250, #V9264, Sigma). Filamentous (F)-actin was stained for 1 h using AlexaFluor^488^ Phalloidin (1:2000, ThermoFisher). Then, a secondary donkey anti-mouse IgG Cy3 antibody (1:400, #IR715-165-150, Jackson) was added for 1 h. After staining, cells were washed with PBS, nuclei stained with DAPI (1:2000) for 30 min in the dark before mounting in Mowiol. Fluorescence microscopy was carried out using an Olympus Ix71 inverted fluorescence phase contrast microscope with quantification done for 10 areas per coverslip containing approximately 130 cells. Macrophages displaying more than five podosomes were counted as podosome positive. For visualization of podosomes by confocal microscopy, a laser scanning LSM 700 (Zeiss) was used.

### Statistics

For statistical analysis, one-way ANOVA, two-tailed unpaired or paired Student’s *t*-test, ratio paired Student’s *t*-test as well as Mann–Whitney test were used as indicated in the figure legends. Multiple testing was corrected using Bonferroni adjustment. For post hoc analysis of time responses Fisher’s LSD multiple comparison test was used. *P*-values below 0.05 were regarded to indicate statistical significance. Graphs were produced and statistical results calculated using GraphPad Prism 8. Boxplots indicate median ± interquartile range, while whiskers show minimum and maximum. Bar graphs show individual data points with means ± standard deviations.

### Data availability

The raw data and count tables used in RNA-seq analysis are available through NCBI’s GEO repository under accession GSE217140. The R-markdown file used for the analysis of RNA-seq data presented in this paper is available in the repository: https://github.com/JureFabjan/macrophage_plasticity.

## Results

### Intrinsically elevated VLCFAs due to ABCD1 deficiency prime the macrophage cell membrane for pro-inflammatory response

In X-ALD, the impaired ability of macrophages to degrade saturated VLCFAs results in exaggerated pro-inflammatory gene expression upon LPS stimulation [12]. To uncover the mechanism by which saturated VLCFAs promote the pro-inflammatory response, we first applied whole transcriptome sequencing to assess how excessive VLCFA levels modulate the activation state of X-ALD macrophages. To avoid strong extrinsic pro-inflammatory cues associated with the onset of CALD that might cover subtle changes associated with intrinsic VLCFA accumulation in X-ALD cells, we included only X-ALD patients who had not developed cerebral involvement at the time of blood donation for this study.

Therefore, we isolated CD14+ monocytes from peripheral blood of nine adult male X-ALD patients lacking signs of brain inflammation and nine age and sex-matched healthy controls. The purity of the cells was determined by flow cytometry (Additional file 1: Fig. S1). The isolated monocytes were differentiated in vitro to homeostatic macrophages in the presence of M-CSF for 7 days before RNA-sequencing was performed. Pathway enrichment analysis revealed the most significant differences between X-ALD and control macrophages for the mRNA levels of genes related to the inflammatory response (Table 1).

**Table 1:** Top five differentially regulated canonical pathways in X-ALD macrophages

Term	Overlap	*p* value	Adjusted *p* value
Inflammatory response (GO:0006954)	78/230	2.59E−07	0.001396
Regulation of T cell proliferation (GO:0042129)	33/76	2.08E−06	0.005601
L-serine metabolic process (GO:0006563)	9/10	3.68E−06	0.00659
Cholesterol biosynthetic process (GO:0006695)	19/35	5.84E−06	0.006709
Sterol biosynthetic process (GO:0016126)	20/38	6.24E−06	0.006709

Top 5 enriched GO Biological Processes with genes found to be differentially expressed between macrophages derived from X-ALD patients and healthy controls (*n* = 9 each). Adjusted *p*-values were calculated using Benjamini and Hochberg method

**Fig. 1:**
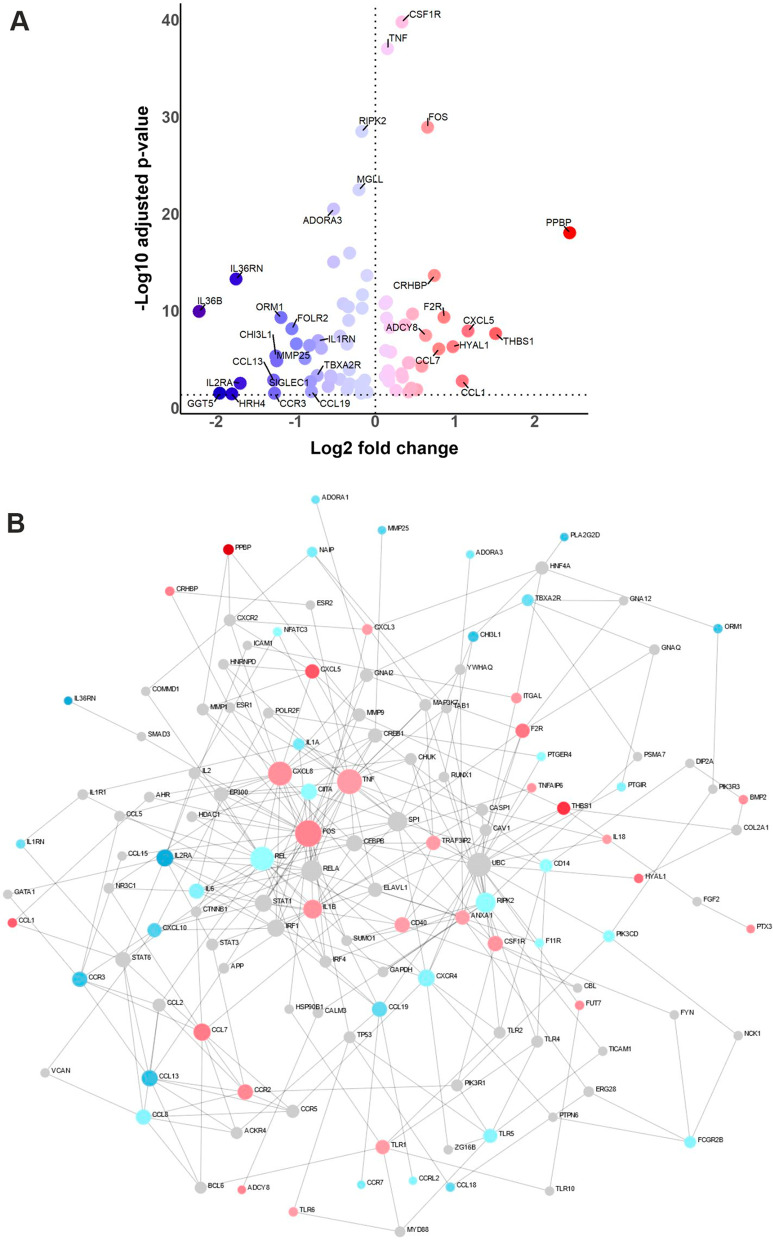
RNA-seq transcriptional profiling of X-ALD macrophages reveals alterations in genes associated with the immune response. **A** Volcano plot depicting differentially expressed genes belonging to the inflammatory response Gene Ontology Term (GO:0006954) of macrophages, derived by in vitro differentiation of monocytes from X-ALD patients compared to healthy controls (*n* = 9 for each). Red-coloured dots represent upregulated genes, whereas downregulated genes are indicated in blue. The fold change and the adjusted *p*-values are indicated on a log2 and log10 scale, respectively. Differentially expressed genes with either log2 fold changes higher than ± 0.6 or − log10 adjusted *p*-values higher than 18 were tagged with the indicated gene symbol. **B** Interactome graph of the inflammatory response Gene Ontology Term (GO:0006954) of macrophages derived from X-ALD patients compared to healthy controls (*n* = 9 for each). Nodes depict genes, while edges show protein–protein interactions between gene products. The colour of nodes depicts log2 fold change, with red indicating upregulated, blue downregulated and grey non-differentially expressed genes. The frequency of protein–protein interaction is reflected by the size of the node

Within this Gene Ontology (GO) category, the top upregulated genes were enriched for membrane receptors and membrane-associated proteins involved in pro-inflammatory signalling, such as *C–C* *chemokine* *receptor* *type* *2* (*CCR2*) and *F2R* (*protease* *activated* *receptor* *1*, *PAR1*), encoding two G-protein coupled receptors, and *thrombospondin* *1* (*THBS1*). CCR2 mediates the transmigration of immune cells across brain endothelial cells during neuroinflammation [22–26], while F2R primes macrophages for LPS and IFNγ responses [27]. THBS1 is a protein with anti-angiogenic properties, which is induced by saturated fatty acids and interacts with the fatty acid translocase CD36 to stimulate TLR4 signalling and monocyte/macrophage adhesion to the blood–brain barrier (BBB) [28, 29] (Fig. 1, Additional file 1: Fig. S2).

We also observed the induction of genes encoding secreted pro-inflammatory mediators, mainly chemokines, such as *CXCL7* (also named *pro-platelet* *basic* *protein*, *PPBP*), *CLL7*, *CXCL5* and *CXCL8*. CXCL7/PPBP is a fatty acid-induced chemokine elevated in atherosclerosis; CCL7 is a macrophage foam cell marker [30] recruiting monocytes to sites of inflammation and has a high affinity to CCR2. CXCL5 is a chemokine regulating macrophage cholesterol efflux as well as tissue remodelling/invasion. CXCL8 is a chemotactic factor for both neutrophils and macrophages that modulates cell adherence and formation of membrane protrusions but also acts on BBB permeability for immune cell infiltration [31, 32]. Furthermore, X-ALD macrophages had elevated expression of the pro-inflammatory cytokines *tumour* *necrosis* *factor* *alpha* (*TNF*) and *interleukin* *1* *beta* (*IL1B*) and of the transcription factor *c-FOS*. As a heterodimer with c-JUN, cFOS constitutes AP-1, which is a transcription factor activated by the c-Jun N-terminal kinase (JNK) pathway linked to stress signals and migration. In contrast to the upregulated mRNAs encoding proteins involved in pro-inflammatory reactions, we found downregulated expression of factors attenuating the pro-inflammatory response of macrophages including: *Interleukin* *36* *receptor* *antagonist* (*IL36RN*), *Interleukin* *36* *beta* (*IL36B*), *Alpha-1-acid* *glycoprotein* *1* (*ORM1*), *Chitinase* *3-like* *1* (*CHI3L1*) and *Phospholipase* *A2* *Group* *IID* (*PLA2G2D*, Fig. 1, Additional file 1: Fig. S2).

When we visualized the functional interaction of the differentially expressed genes within the GO Term Inflammatory Response using the NetworkAnalyst software, we observed that the top three interaction nodes were CXCL8, TNF and FOS (Fig. 1B). Together, our results from whole transcriptome RNA-sequencing of X-ALD macrophages versus controls indicate that saturated VLCFAs set up the macrophage plasma membrane for pro-inflammatory activity, while simultaneously repressing factors regulating the balance between pro- and anti-inflammatory macrophage responses.

### Saturated VLCFAs do not directly activate TLR signalling but propagate the pro-inflammatory macrophage response by stimulating the CD36–JNK axis

**Fig. 2:**
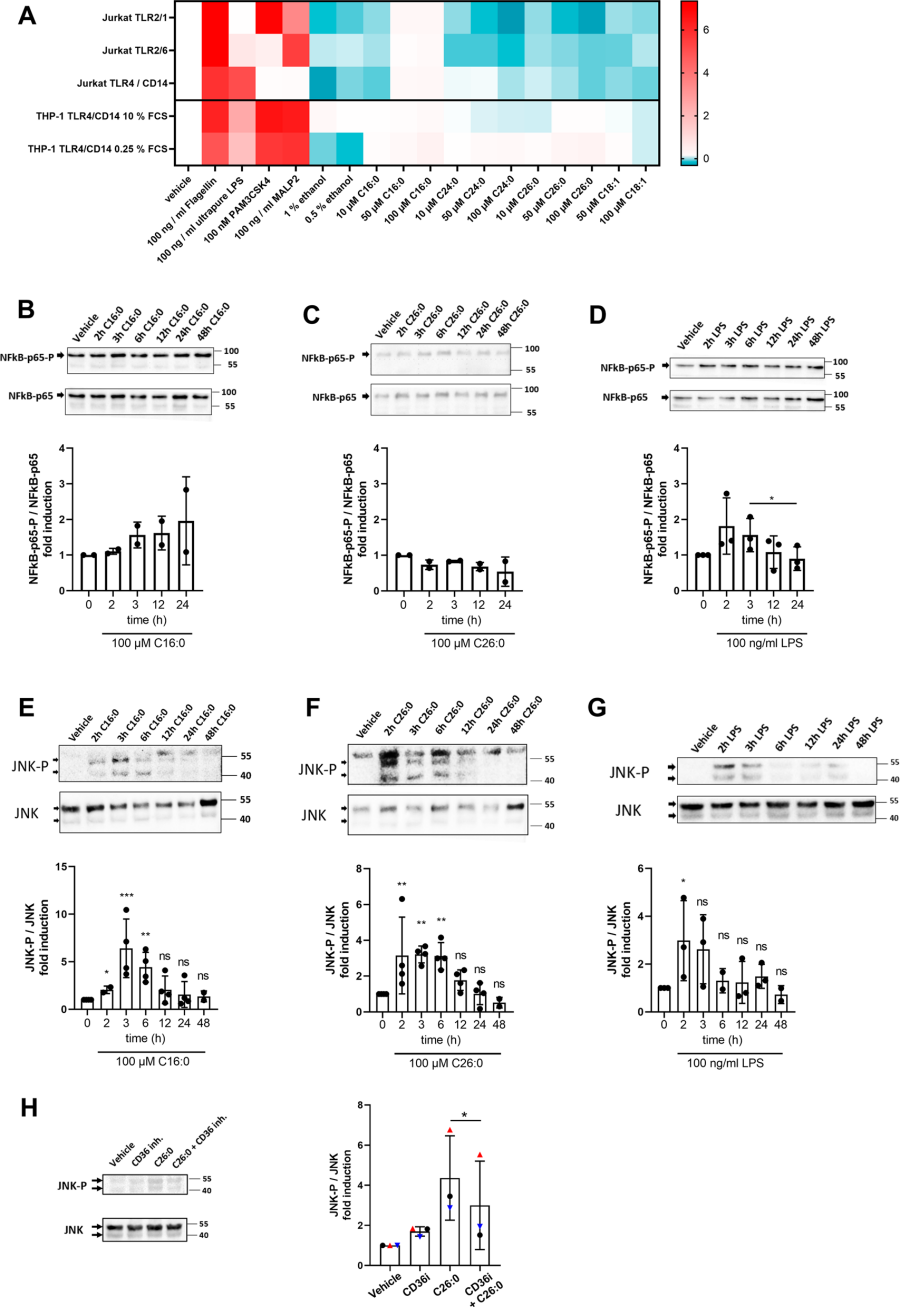
The saturated VLCFA C26:0 activates the JNK stress kinase pathway but not TLR-mediated NFκB signalling. **A** Reporter Jurkat E6-1-NFκB::eGFP-TLR2/1, Jurkat E6-1-NFκB::eGFP-TLR2/6 and Jurkat E6-1-NFκB::eGFP-TLR4/CD14 cells, as well as reporter THP-1 E6-1-NFκB::eGFP-TLR4/CD14 cells were incubated with either the cognate TLR ligands flagellin, LPS, PAM3CSK4 or MALP2 or saturated LCFA (C16:0), saturated VLCFAs (C24:0, C26:0) or mono-unsaturated LCFA (C18:1) as indicated. After 24 h, eGFP expression was assessed by flow cytometry. The heatmap represents mean fold change to vehicle of 2 replicates. **B**–**G** Primary human macrophages derived from healthy control donors (*n* = 2–4) were treated with either the solvent ethanol (vehicle), C16:0 (100 µM), C26:0 (100 µM) or LPS (100 ng/ml) for the indicated time. Immunoblotting was performed on cell lysates analysing the levels of **B**–**D** phosphorylated and total NFκB-p65 or **E**–**G** phosphorylated and total JNK1 (46 kDa)/JNK2 (55 kDa). **H** Macrophages derived from 3 healthy control donors were incubated with either C26:0 (100 µM), the CD36 inhibitor (CD36i) sulfosuccinimidyl oleate (100 µM) or both compounds for 24 h prior to immunoblotting for phosphorylated and total JNK1/JNK2. Values are shown as the mean fold change to vehicle control and error bars indicate standard deviation. One-way ANOVA and Fisher’s LSD multiple comparison test were performed in **B**–**G**. Paired two-way Student’s *t*-test was performed in **H**. *p < 0.05; **p < 0.01; ***p < 0.001; *ns* not significant

Saturated VLCFAs are particularly enriched in the brain, where these fatty acids function in decreasing myelin fluidity and providing a permeability barrier to insulate axons [33]. With onset of neuroinflammation and associated myelin degradation, VLCFA-enriched lipids strongly accumulate in CALD brain lesions, where they constitute up to 67% of fatty acids in cholesterol esters [34]. Thus, we next assessed how extracellular saturated VLCFAs act on macrophage activation. We first investigated whether C24:0 and C26:0, the VLCFAs most prominently accumulating in X-ALD, are recognized by the toll-like receptors TLR4, TLR2/1 or TLR2/6, which are endogenously expressed on the surface of macrophages. To test this hypothesis, we made use of modified Jurkat T cells, which endogenously express solely TLR5 but were engineered to overexpress either TLR4 homo- or TLR2/1 or TLR2/6 heterodimers together with an NFκB-eGFP reporter plasmid for downstream detection of NFκB signalling [14]. In this assay, only the recognition of a matching ligand by the TLRs induces dimerization and internalization, leading to an NFκB-mediated downstream fluorescent signal that can be detected and quantified by flow cytometry. Using this reporter system, we found that exposure to different concentrations of C24:0 and C26:0 did not initiate a NFκB response (Fig. 2A), whereas cognate binding partners for each TLR receptor (flagellin, LPS, PAM3CSK4 and MALP2) induced NFκB signalling (Fig. 2A).

In addition, neither treatment with the saturated LCFA C16:0 nor with the mono-unsaturated LCFA C18:1 activated TLR signalling, thus confirming previous investigations [35]. To further evaluate these findings in myeloid cells naturally equipped with TLR4, TLR2/1 and TLR2/6 receptors, we treated a monocytic THP-1 cell line stably expressing NFκB-eGFP [15] with either the fatty acids or the cognate ligands. However, also in these cells, neither the saturated VLCFAs C24:0 and C26:0 nor the LCFAs C16:0 and C18:1 were able to activate the NFκB pathway, despite clear positivity resulting from flagellin, LPS, PAM3CSK4 or MALP2 treatment (Fig. 2A). We finally confirmed our findings in primary human macrophages by Western blot analysis after exposure to C26:0, C16:0 or LPS for up to 24 h. In contrast to LPS, neither C26:0 nor C16:0 fatty acids induced activation of the NFκB pathway, as evidenced by absent phosphorylation of NFκB-p65 after treatment (Fig. 2B–D). The viability of primary human macrophages was not severely affected by C26:0 treatment (Additional file 1: Fig. S3). Collectively, these data show that the saturated VLCFA C26:0 and the saturated LCFA C16:0 are unable to elicit TLR4, TLR2/1 and TLR2/6 signalling in Jurkat T cells, monocytic THP-1 cells and primary monocyte-derived macrophages.

Besides NFkB as a master coordinator of pro-inflammatory macrophage activation, also stimulation of the c-Jun N-terminal kinase (JNK) pathway is key in the induction of inflammatory responses in macrophages including cell migration and cytokine production [36]. Of note, saturated LCFAs but not polyunsaturated fatty acids (PUFAs) were previously identified as relevant JNK activators that induce clustering of the tyrosine kinase c-SRC in membrane subdomains, thereby leading to activation of JNK signalling [3, 37]. Therefore, we next assessed whether C26:0 elicits a pro-inflammatory response in macrophages by activating the JNK pathway. Indeed, Western blot analysis of human macrophages treated with C26:0 for up to 48 h revealed significantly increased JNK phosphorylation (JNK-P) levels that peaked at 3 h and persisted up to 6 h of incubation (Fig. 2F). As expected, both C16:0 and LPS induced JNK phosphorylation (Fig. 2E, G).

Due to their chemical properties, VLCFAs are unable to freely diffuse across the plasma membrane. Thus, we next asked whether the translocation through a protein-based mechanism could be a prerequisite for C26:0-mediated JNK signalling. Previously, the scavenger receptor CD36 was identified to be required for the uptake of saturated VLCFAs in COS-7 cells and absorption of dietary VLCFAs in the intestine of mice [38]. To examine whether CD36 is involved in C26:0 inducing the JNK-pathway in macrophages, we stimulated the cells with C26:0 in the presence of the CD36 competitive inhibitor sulfosuccinimidyl oleate (SSO) [39]. Indeed, inhibition of CD36 by SSO co-treatment significantly reduced activation of the JNK pathway in C26:0-exposed macrophages (Fig. 2H, Additional file 1: Fig. S4). Collectively, these data indicate that C26:0 needs to be transported into macrophages through the fatty acid translocase CD36 to promote intracellular JNK signalling.

### Saturated VLCFA treatment specifically stimulates macrophages for chemokine secretion and upregulation of extracellular matrix-degrading enzymes

**Fig. 3:**
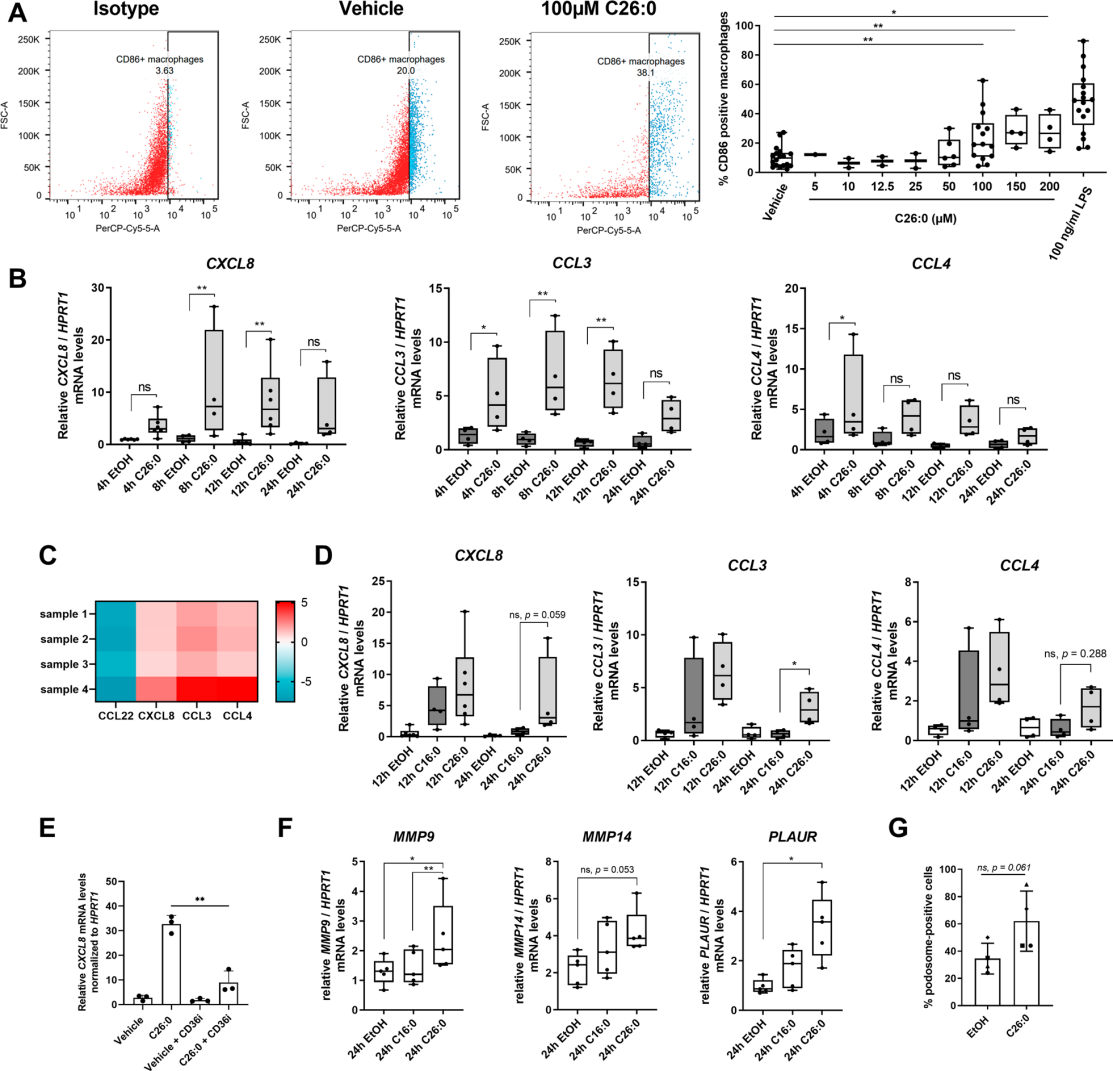
Saturated VLCFA exposure initiates pro-inflammatory chemokine production in human macrophages. **A** Human macrophages derived from healthy control donors (*n* = 14) were treated with different concentrations of C26:0 or LPS for 24 h before expression of the pro-inflammatory cell surface marker CD86 was assessed by flow cytometry. **B** Human healthy control macrophages (*n* = 4–6) were treated with C26:0 (100 µM) or the solvent EtOH for the indicated time. RT-qPCR was carried out to measure mRNA levels of *CXCL8*, *CCL3* and *CCL4*, and normalized to *HPRT1*. **C** Healthy control macrophages (*n* = 4) were treated with C26:0 (100 µM) or the solvent EtOH for 24 h. Supernatants were analysed for CXCL8, CCL3 and CCL4 protein levels using Luminex ELISA bead assays. The heat map indicates fold changes to solvent-treated samples. **D** RT-qPCR of *CXCL8*, *CCL3* and *CCL4* mRNA levels were normalized to *HPRT1* in macrophages (*n* = 4) treated with either C16:0 (100 µM) or C26:0 (100 µM) for 12 or 24 h. **E** RT-qPCR of *CXCL8* expression in healthy control macrophages (*n* = 3) treated with C26:0 (100 µM) or the CD36 inhibitor (CD36i) sulfosuccinimidyl oleate (100 µM) or with both compounds for 24 h. **F** RT-qPCR of *MMP9*, *MMP14* and *PLAUR* normalized to *HPRT1* was performed in healthy control macrophages (*n* = 5) treated with either C16:0 (100 µM) or C26:0 (100 µM) for 24 h. **G** Healthy control macrophages derived from 4 donors were treated with C26:0 (100 µM) or the solvent EtOH for 24 h before podosome structures were visualized by staining f-actin with AlexaFluor^488^-phalloidin and the frequency of podosome-positive macrophages being determined by fluorescence microscopy. One-way ANOVA and Fisher’s LSD multiple comparison test were performed in **A** and the ratio paired *t*-test was used in **B**, **D**–**G**: **p < 0.01; *p < 0.05; *ns* not significant. Boxplots indicate median ± interquartile range, while whiskers show minimum and maximum. Bar graphs show means ± standard deviations

Having shown that CD36-mediated uptake of C26:0 stimulates the JNK pathway, we next assessed how this activation impacts pro-inflammatory responses in healthy human macrophages. Using FACS analysis, we found that incubation of macrophages with C26:0 increased the expression of CD86 (Fig. 3A), a surface activation marker previously identified to be upregulated on macrophages/microglial cells in active brain lesions of CALD patients [12].

In contrast, no significant modulation of the expression of classical pro-inflammatory cytokine genes such as *IL6*, *TNF* or *IL1B* was observed (Additional file 1: Fig. S5). To test whether VLCFA treatment promotes chemokine production, as suggested by the whole transcriptome analysis of X-ALD macrophages (Fig. 1), we used RT-qPCR to analyse the expression of *CXCL8*, *CCL3* and *CCL4* in macrophages treated with C26:0 for up to 24 h. We found that C26:0 significantly stimulated the expression of all three investigated chemokines (Fig. 3B). To further confirm the production of these chemokines at the protein level, we collected the supernatants of control macrophages incubated in the presence of C26:0 for 24 h and performed multiplex Luminex bead-based immunoassay for CXCL8 (IL-8), CCL3 (MIP-1α) and CCL4 (MIP-1β) and additionally for CCL11 (eotaxin), CX3CL1 (fraktaline), CXCL1 (GRO), CCL7 (MCP-3), IL-12p40, CCL22 (MDC), IL-15, CXCL10 (IP-10) and CCL2 (MCP-1) (Fig. 3C, Additional file 1: Fig. S6). We found a striking increase for the release of CXCL8 and CCL3 and a tendency towards elevated secretion of CCL4 upon C26:0 treatment (Fig. 3C). In contrast, VLCFA supplementation reduced the levels of CCL22 (Fig. 3C), CXCL10 and CCL7, whereas CCL2 and CXCL1 secretion was not affected (Additional file 1: Fig. S6). In this assay, we were unable to detect CCL11, CX3CL1, IL-12p40 and IL-15 in the macrophage supernatants both before and after VLCFA treatment. As C16:0 treatment was previously shown to impact *CXCL8* expression [40], we next compared the effects of C26:0 and C16:0 on *CXCL8*, *CCL3* and *CCL4* gene expression. We found that incubation with C26:0 for 24 h resulted in significantly higher *CCL3* mRNA levels and a trend towards increased *CXCL8* and *CCL4* expression when compared to C16:0 (Fig. 3D). We also assessed whether the production of chemokines in response to VLCFAs involves the CD36 fatty acid translocase by co-incubating macrophages with C26:0 and the CD36-inhibitor SSO. Our data revealed that inhibiting CD36 activity significantly interfered with C26:0 mediated upregulation of *CXCL8* mRNA levels, indicating that CXCL8 chemokine production in response to VLCFAs is CD36-dependent (Fig. 3E).

In X-ALD macrophages, the lack of ABCD1 with associated VLCFA accumulation is not only linked to chemokine upregulation, but also to increased expression of genes associated with BBB adhesion and invasion (Fig. 1). Therefore, we also tested whether treatment of healthy control macrophages with either C26:0 or C16:0 for 24 h alters the expression of genes responsible for the proteolytic degradation of extracellular matrix molecules at the basal lamina of the BBB. The mRNA levels analysed included genes encoding matrix metalloproteinases (*MMP9* and *MMP14*) as well as the urokinase plasminogen activator surface receptor (*PLAUR*), which induces extracellular matrix degradation through protease activity. We found that C26:0 significantly induced the expression of *MMP9* and *PLAUR* and tendentially (*p* = 0.053) increased *MMP14* mRNA levels after 24 h of treatment (Fig. 3F). Concomitant with these results, we observed that C26:0 treatment increased the frequency of macrophages presenting podosomes (Fig. 3G, Additional file 1: Fig. S7), which are dynamic adhesive structures involved in extracellular matrix degradation through recruitment of matrix lytic enzymes such as metalloproteinases.

### Macrophages remodel their VLCFA content in response to inflammation

**Fig. 4:**
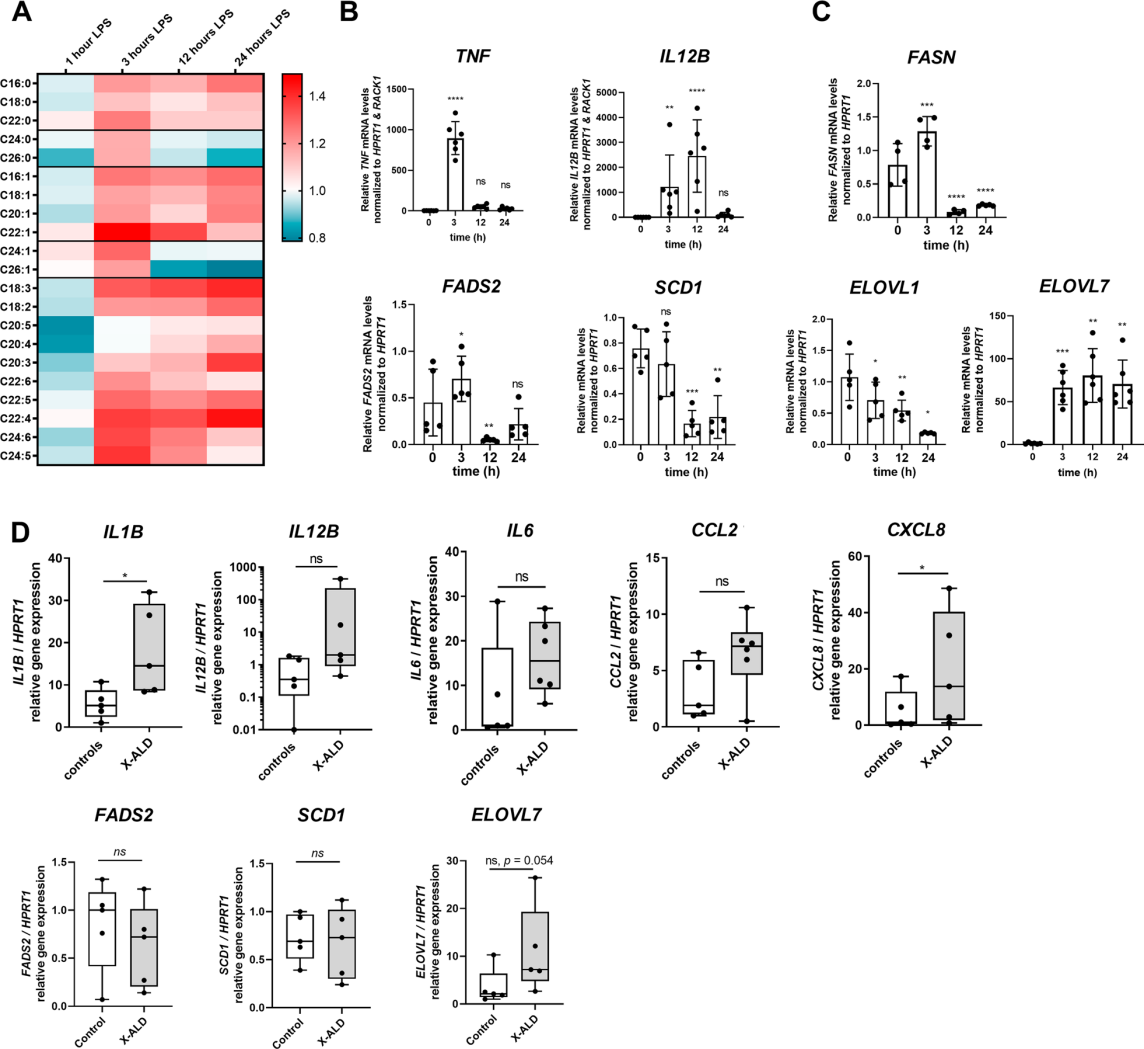
Macrophages modulate their saturated VLCFA content according to their activation state. **A** The levels of saturated and mono-unsaturated LCFAs and VLCFAs, as well as PUFAs of human macrophages incubated with LPS for 1, 3, 12 or 24 h were determined by ESI–MS. The heatmap represents mean values of macrophages derived from 5 healthy donors. RT-qPCR analysis of healthy control macrophages incubated with LPS for 1, 3, 12 or 24 h shows gene expression of **B** acute pro-inflammatory markers (*TNFA*, *IL12B*) as well as **C** enzymes involved in fatty acid synthesis (*FASN*, *FADS2*, *SCD1*, *ELOVL1*, *ELOVL7*). Data were normalized to *HPRT1* and *RACK1* or *HPRT1* mRNA levels. **D** Macrophages derived from X-ALD patients (*n* = 5–7) and healthy controls (*n* = 5–7) were incubated with LPS for 24 h. RT-qPCR was carried out to assess expression of pro-inflammatory markers (*IL1B*, *IL12B*, *IL6*, *CCL2* and *CXCL8*) and enzymes involved in fatty acid synthesis (*FADS2*, *SCD1* and *ELOVL7*). For statistical analysis one-way ANOVA and Fisher’s LSD multiple comparison test were performed in **B**, **C** and the Mann–Whitney test was used in **D**: ***p < 0.001; **p < 0.01; *p < 0.05; *ns* not significant. Boxplots indicate median ± interquartile range, while whiskers show minimum and maximum. Bar graphs show means ± standard deviations

Treatment of macrophages with the saturated VLCFA C26:0 promotes JNK signalling and cumulates in the release of pro-inflammatory mediators, mainly chemokines. In addition, our RNA-seq data suggest that VLCFA accumulation in X-ALD macrophages predisposes them for pro-inflammatory responses. Based on these observations we hypothesized that macrophages need to tightly regulate their intracellular VLCFA levels to prevent exaggerated inflammatory responses. To test this notion, we analysed how the cellular VLCFA content changes with the onset and duration of pro-inflammatory stimulation. We applied electrospray ionization mass spectrometry (ESI–MS) to determine the fatty acid profile after hydrolysis of fatty acyl esters, which provides a good estimation of membrane lipid abundance of macrophages during different stages of pro-inflammatory activation. We found that upon LPS treatment of healthy control macrophages, saturated and mono-unsaturated VLCFAs (C24:0 and C26:0 as well as C24:1 and C26:1) increased during the immediate/early pro-inflammatory response, when acute mediators like TNFα are produced (up to 3 h after LPS addition, Fig. 4A, B).

The amplification of VLCFA levels was paralleled by a general increase in saturated and mono-unsaturated LCFAs as well as PUFAs, thus confirming previous reports [3]. The elevation in macrophage fatty acid content was reflected in the upregulation of enzymes involved in de novo biosynthesis of saturated LCFAs (*fatty* *acid* *synthetase*, *FASN*) and PUFAs (*fatty* *acid* *desaturase* *2*, *FADS2* and *elongation* *of* *very* *long-chain* *fatty* *acids* *7*, *ELOVL7*) at 3 h post-LPS application (Fig. 4C). The levels of *stearoyl-CoA* *desaturase* (*SCD1*, *alias* *FADS5*), responsible for the conversion of saturated LCFAs to mono-unsaturated LCFAs, remained unchanged in the early inflammatory state (Fig. 4C). With the onset of resolution of the pro-inflammatory state (pro-resolution) and decreasing expression of the acute mediator *TNF* and peak expression of the late response gene *IL12B* (12 h post-LPS addition, Fig. 4B), our data revealed a selective reduction of saturated and mono-unsaturated VLCFAs. This was accompanied by significant downregulation of *ELOVL1*, encoding the enzyme involved in saturated VLCFA synthesis [41] (Fig. 4A, C). In contrast, saturated and mono-unsaturated fatty acids with shorter chain lengths (≤ C22) or highly unsaturated VLCFAs (C24:6 and C24:5) still remained elevated 24 h post-LPS application, despite preceding downregulation of enzymes involved in their synthesis (*FASN*, *FADS2* and *SCD1*, Fig. 4C). The expression of *ELOVL7*, which is required for synthesis of anti-inflammatory PUFAs, remained upregulated during the resolution phase (Fig. 4C).

In X-ALD macrophages, VLCFA accumulation combined with an impaired ability to remodel their elevated VLCFA content prolonged the expression of acute pro-inflammatory mediator genes when compared to healthy control cells at 24 h post-LPS application (Fig. 4D), thus confirming our previous results [42–44]. Of note, the increased pro-inflammatory gene expression in X-ALD macrophages upon LPS-treatment was also reflected by a trend towards elevated *ELOVL7* expression, an enzyme strongly upregulated with LPS-treatment (Fig. 4C), when compared to control cells (Fig. 4D). Regarding *FADS2* and *SCD1*, X-ALD cells were able to downregulate the expression of these genes to similar extents as controls (Fig. 4D). Together, our results demonstrate that macrophages quickly metabolize saturated and mono-unsaturated VLCFAs with transition from the pro-inflammatory phase to the onset and establishment of resolution. In ABCD1-deficient (X-ALD) macrophages with impaired catabolism of VLCFAs, the excess of VLCFAs impacts the plasticity of macrophages by prolonging the pro-inflammatory response.

### The rate of peroxisomal VLCFA degradation is linked to the pro-inflammatory status of macrophages

**Fig. 5:**
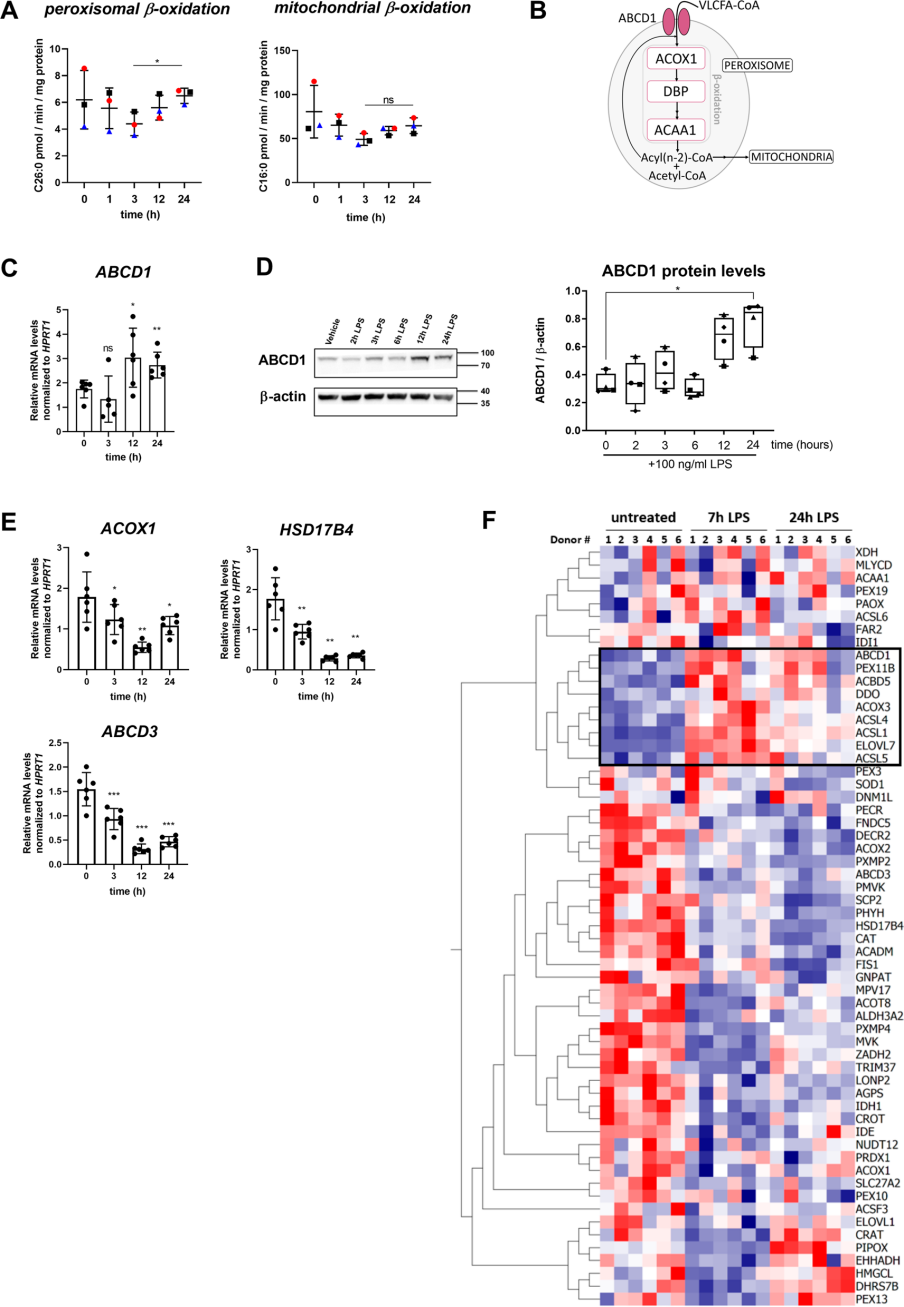
Saturated VLCFAs are degraded by peroxisomal β-oxidation with onset of pro-inflammatory resolution. Macrophages derived from healthy control donors were treated with LPS and incubated for the indicated time. **A** The mean values of C26:0 and C16:0 degradation by peroxisomal and mitochondrial β-oxidation normalized to protein content are shown (*n* = 3). **B** Scheme indicating the enzymes involved in peroxisomal β-oxidation (acyl-coenzyme A oxidase 1, ACOX1; D-bifunctional protein, DBP; acetyl-CoA acyltransferase 1, ACAA1). **C** RT-qPCR of *ABCD1* normalized to *HPRT1* mRNA levels (*n* = 6). **D** Immunoblot analysis to determine ABCD1 protein levels normalized to β-actin of macrophages derived from 4 healthy donors. Representative blot shows values from one healthy donor. **E** RT-qPCR of *ACOX1*, *HSD17B4* and *ABCD3* expression with normalization to *HPRT1* mRNA levels (*n* = 6). **F** Microarray data from LPS-stimulated human primary macrophages (*n* = 6) were retrieved from Regan et al., (GSE85333) and log 2-fold changes are shown by the heatmap. One-way ANOVA and Fisher’s LSD comparison test was used for statistical analysis in **A**, **C**–**E**. Bar graphs indicate means ± standard deviations. ***p < 0.001; **p < 0.01; *p < 0.05; *ns* not significant

Our results suggest that macrophages must clear VLCFAs to efficiently terminate the pro-inflammatory response. As VLCFAs are degraded by β-oxidation within peroxisomes, we next asked how the peroxisomal β-oxidation activity of macrophages responds to LPS treatment. Therefore, we analysed the VLCFA degradation rate by peroxisomes and, for comparison, the rate of breakdown of LCFAs by mitochondrial β-oxidation in LPS-stimulated primary human macrophages at the onset and up to 24 h post-application of the pro-inflammatory stimulus. The degradation of VLCFAs requires the activation of the fatty acids through addition of coenzyme A and transport into the organelles. In the peroxisomal matrix, the β-oxidation, consisting of dehydrogenation, hydration, oxidation and thiolytic cleavage, occurs resulting in the shortening of the acyl-CoA chain by two carbons per cycle (Fig. 5B).

In the in vitro β-oxidation assay, this release of water-soluble C_2_ units from radioactively labelled C26:0 or C16:0 is measured. Accompanying the general increase in fatty acid content early upon LPS-stimulation (Fig. 4A), we observed tendentially lowered rates for both peroxisomal and mitochondrial β-oxidation rates accompanying pro-inflammatory activation in macrophages (Fig. 5A). However, with the onset of pro-inflammatory resolution, aimed to terminate the pro-inflammatory response (24 h post-addition of LPS), only the peroxisomal fatty acid β-oxidation was significantly elevated, thus returning to pre-treatment levels (Fig. 5A).

To elucidate whether the increased peroxisomal β-oxidation upon entering the pro-inflammatory resolution phase is caused by a concerted upregulation of peroxisomal genes involved in VLCFA degradation, we treated macrophages with LPS and harvested cells at 3, 12 and 24 h post-LPS application. We assessed expression of *ABCD1*, encoding the rate-limiting VLCFA importer, as well as of the peroxisomal β-oxidation enzymes, *acyl-CoA* *oxidase* *1* (*ACOX1*) and *hydroxysteroid* *17-beta* *dehydrogenase* *4* (*HSD17B4*, encoding D-bifunctional protein, DBP) (Fig. 5B). We found that ABCD1 was significantly upregulated starting at 12 h post-application of LPS at both mRNA and protein level (Fig. 5C, D), thus being induced concurrently with peroxisomal β-oxidation activity at resolution. Intriguingly, our analysis revealed that within the acute pro-inflammatory response (3 h post-LPS addition), expression of both *ACOX1* and *HSD17B4* were significantly downregulated in macrophages (Fig. 5E). We also tested whether *ABCD3*, encoding another peroxisomal fatty acid transporter that imports long-chain unsaturated-, long branched-chain- and long-chain dicarboxylic fatty acids for peroxisomal degradation, is modulated by pro-inflammatory stimulation and thus, would contribute to the increased fatty acid levels in activated macrophages. Indeed, *ABCD3* was significantly downregulated early upon LPS application and remained repressed at 24 h post-LPS addition, thus further lending support to this concept (Fig. 5E).

To assess how genes involved in LCFA/VLCFA metabolism and peroxisomal genes in general respond to pro-inflammatory activation of macrophages, we retrieved transcriptomics data from the Gene Expression Omnibus (GEO) dataset GSE85333 [45], obtained from macrophages with a similar differentiation and LPS activation protocol as used in our study. By reanalysing this data set, we observed prominent upregulation of *ABCD1*, confirming our own results, and of a subset of genes including long-chain acyl-CoA synthetase (ACSL) family members (*ACSL1*, *ACSL4* and *ACSL5*) involved in CoA-activation of fatty acids, which is a prerequisite for entering metabolic pathways like β-oxidation. Interestingly, most genes encoding peroxisomal proteins, including *ABCD3*, were repressed 7 and 24 h post-LPS addition (Fig. 5F), in line with our data. Collectively, these findings show that after the acute pro-inflammatory response to LPS, with the onset of resolution, macrophages specifically upregulate the VLCFA transporter ABCD1 to enable degradation of VLCFAs within peroxisomes. Despite this induction of ABCD1, the majority of peroxisomal genes including those involved in plasmalogen synthesis (alkylglycerone phosphate synthetase, *AGPS*; glyceronephosphate O-acyltransferase; *GNPAT*; fatty acyl-CoA reductase 2, *FAR2*), were found to be downregulated.

### VLCFA degradation in macrophages during resolution of inflammation involves LXR-mediated upregulation of *ABCD1* expression

**Fig. 6:**
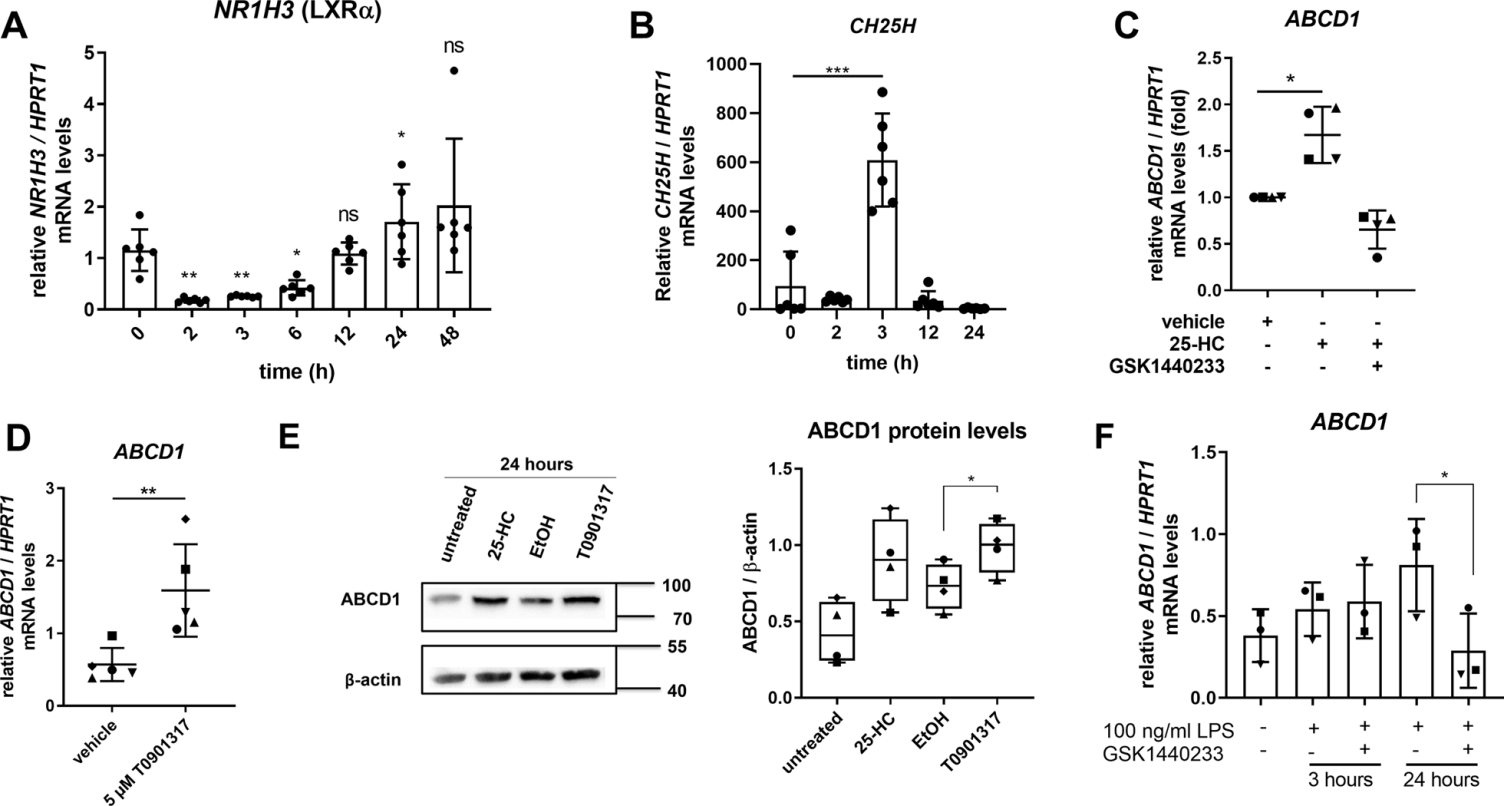
The upregulation of ABCD1 in human macrophages entering pro-inflammatory resolution is mediated by LXRα. **A**, **B** Macrophages derived from healthy control donors were treated with LPS and incubated for the indicated time. The expression of *NR1H3* (encoding LXRα) and *CH25H* normalized to *HPRT1* was analysed by RT-qPCR (*n* = 6). **C** Macrophages from 4 healthy donors were treated with either 25-hydroxycholesterol (25-HC), the LXR antagonist GSK1440233 or the solvent EtOH for 24 h followed by analysis of *ABCD1* expression by RT-qPCR. **D** Macrophages from 5 healthy donors were treated with the LXR-agonist T0901317 or solvent control for 24 h and ABCD1 levels normalized by *HPRT1* were determined RT-qPCR. **E** Immunoblot analysis of macrophages treated with either 25-HC, the LXR-agonist T0901317 or solvent control to determine ABCD1 protein levels normalized to β-actin (*n* = 4). Representative blot of macrophages derived from one healthy donor is shown. **F** Macrophages derived from three healthy donors were treated with either LPS or LPS and the LXR antagonist GSK1440233 for 3 or 24 h before *ABCD1* mRNA levels normalized for *HPRT1* were determined by RT-qPCR. One-way ANOVA and Fisher’s LSD comparison test was used for statistical analysis in **A** and **B**. Ratio paired Student’s *t* test was performed on absolute values in **C**–**E** and paired Student’s *t* test in **F**. ***p < 0.001; **p < 0.01; *p < 0.05; *ns* not significant

As ABCD1 appears to be the molecular switch for quick and selective modification of VLCFA levels in LPS-activated macrophages, we investigated the underlying regulatory mechanism of ABCD1 upregulation at the transition to pro-inflammatory resolution. The nuclear oxysterol receptor and transcription factor liver X receptor (LXRα, also termed NR1H3), a crucial key player in regulating lipid metabolism including cholesterol export, promotes both pro-resolution and anti-inflammatory activity in macrophages [46, 47]. As the onset of efficient resolution of the pro-inflammatory state seems to necessitate clearance of VLCFAs, we hypothesized that *ABCD1* expression could be modulated by LXR. Transcription of LXR itself is downregulated during the acute pro-inflammatory response and upregulated with the start of resolution [3], also in our paradigm (Fig. 6A).

The activation of LXR target genes is triggered by the binding of oxysterol ligands such as 25-hydroxycholesterol (25-HC), which is produced by the enzyme cholesterol-25-hydroxylase (CH25H). Thus, we first determined whether upregulation of *ABCD1* occurs subsequent to 25-HC production by CH25H in our experimental conditions. Our analysis revealed a robust activation of *CH25H* expression at 3 h post-LPS application (Fig. 6B), thus preceding *ABCD1* induction and confirming previous investigations [48]. Addition of 25-HC, the product of CH25H, to the cell culture medium, resulted in significantly upregulated *ABCD1* mRNA levels in 25-HC-stimulated human macrophages (Fig. 6C). To confirm that the 25-HC-mediated induction of ABCD1 involves LXR activity, we added the synthetic LXR antagonist GW1440233, which upon co-treatment abrogated 25-HC-induced *ABCD1* expression (Fig. 6C). To investigate this interaction in more detail, we also applied the synthetic LXRα agonist T0901317 and, indeed, detected a significant upregulation of ABCD1 mRNA and protein levels by RT-qPCR and Western blot analysis, respectively (Fig. 6D, E). Consistent with our hypothesis that LXR mediates the upregulation of *ABCD1* with the start of resolution, we observed that the LXR antagonist GW1440233 abolished induction of *ABCD1* in LPS-treated macrophages (Fig. 6F). Together, our data demonstrate that LXR-mediated induction of *ABCD1* expression enables peroxisomal VLCFA degradation in macrophages entering the pro-resolution state (Fig. 6G).

## Conclusions

**Fig. 7:**
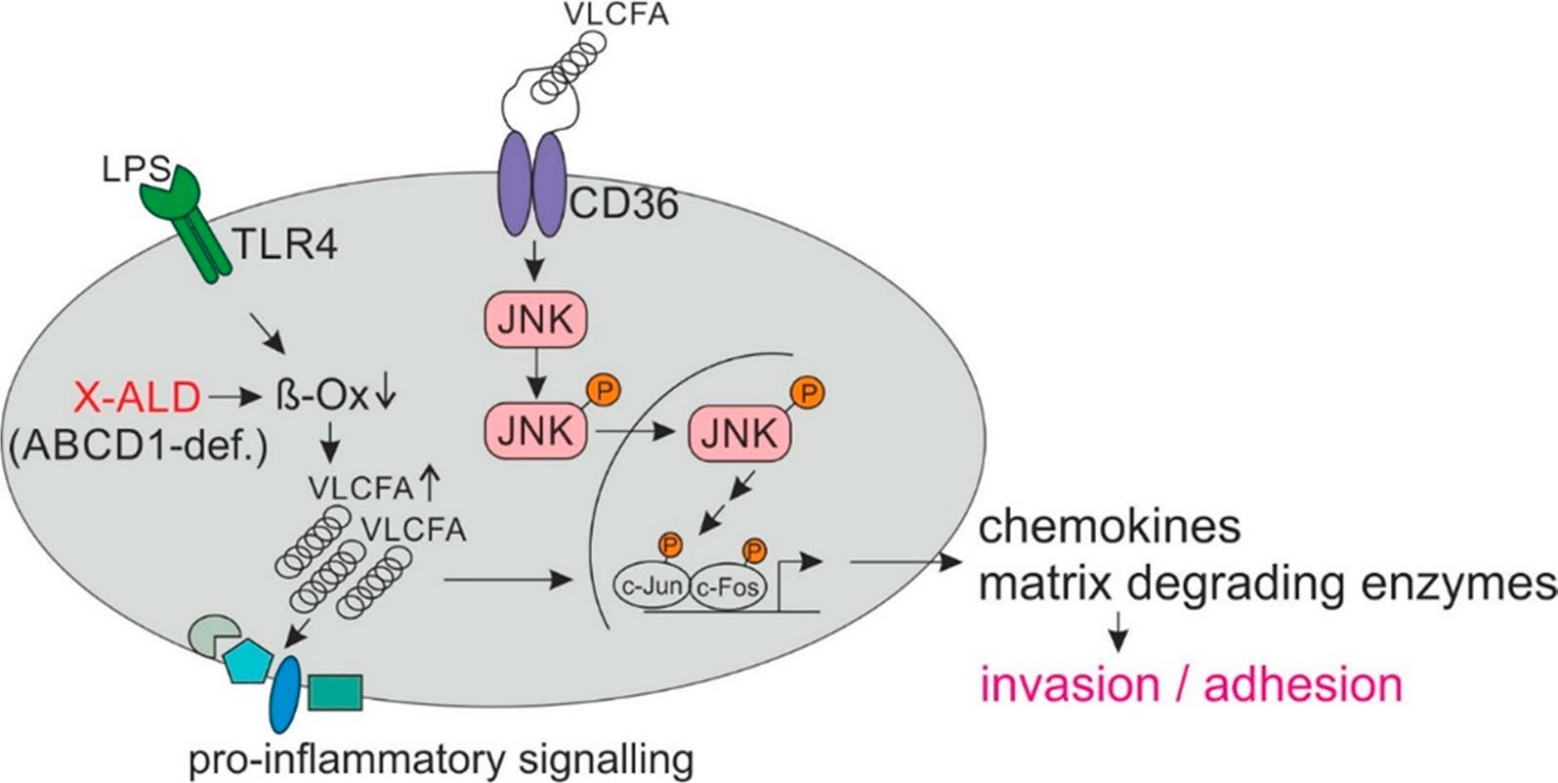
Proposed mechanism for how VLCFAs promote a pro-inflammatory and pro-invasive phenotype of human macrophages. In an acute pro-inflammatory response, macrophages react by increasing the levels of saturated VLCFAs to create a receptive environment in the plasma membrane that enables pro-inflammatory signalling and the production of factors required for invasion and adhesion. When applied externally to mimic the condition in acute cerebral X-ALD lesions, VLCFAs activate the CD36/JNK axis, thus also promoting a pro-inflammatory pro-invasive macrophage response culminating in the secretion of chemokines and matrix-degrading enzymes

Changing the cellular lipid composition has been proposed as a prerequisite for macrophages to shape their immune response to environmental conditions [49]. Here, we provide further evidence for this concept by demonstrating a reciprocal relationship between ABCD1-mediated peroxisomal degradation of saturated VLCFAs and the resolution of pro-inflammatory gene expression in macrophages. Thus, our findings directly link saturated VLCFA levels with a pro-inflammatory, pro-invasive phenotype (Fig. 7). Further, these results lend support to the idea that pharmacological modulation of these fatty acids in monocytes, macrophages and probably microglial cells could be beneficial towards altering the exaggerated innate immune response associated with neuroinflammation in X-ALD patients. Of note, the LPS-mediated upregulation of ABCD1, which is the rate-limiting factor in peroxisomal VLCFA degradation [50], was concurrent with repression of most peroxisomal genes including those involved in β-oxidation. This is the opposite pattern of that observed upon herpesvirus infection of B cells, where pathogen-induced downregulation of ABCD1 occurs despite general induction of peroxisomes [51]. This would allow both accumulation of VLCFAs, which are needed by the virus, and exploitation of other peroxisomal functions required for viral replication [51]. Accordingly, ABCD1 could represent a dynamic regulatory switch point in immune cells either promoting or preventing VLCFA accumulation to modulate pro-inflammatory responses associated with the defence against pathogens.

Saturated fatty acids are thought to create a receptive environment within the plasma membrane that enables the assembly of cholesterol-dependent pro-inflammatory signalling networks [52]. Consistently, our whole-transcriptome analysis of X-ALD macrophages revealed that elevation of saturated VLCFAs primarily increased the expression of membrane receptors and membrane-associated proteins functioning in the pro-inflammatory response. In this regard, the membrane ordering property of saturated VLCFAs could promote sphingolipid- and cholesterol-rich lipid nanodomains and, thus, culminate in the formation of so called inflammarafts [53]. By incorporating ion channels, membrane proteins and enzymes involved in pro-inflammatory signalling, these specialized clustered membrane structures serve as an organizing platform for active molecule complexes such as TLR dimers [54]. Accordingly, inflammarafts are thought to enable downstream signal transduction in macrophages upon recognition of danger signals [54]. A typical characteristic of inflammarafts is the increased cholesterol content per raft. In this respect, it is notable that next to the inflammatory response, also cholesterol and sterol biosynthesis were among the top dysregulated pathways in X-ALD macrophages. Aside from being involved in membrane organization, saturated fatty acids are also increasingly recognized for their roles in protein lipidation. Recently, it was shown that the reversible attachment of C16:0 (palmitoyl-group) to proteins including CD36 stimulates macrophage responses linked to intracellular signalling or chemotaxis [55]. Importantly, next to palmitoylation, also saturated VLCFAs are used for protein acylation, as shown for processes associated with inflammatory programmed cell death, where limiting the amount of VLCFAs decreased membrane recruitment of proteins involved in necroptosis [56]. Accordingly, the identity of the added acyl chain, such as C16:0 or C26:0 with different affinities for membranes and cholesterol-rich lipid plasma membrane nanodomains [57], could be central to the regulatory effects of lipidation of proteins and in fine tuning macrophage responses to specific danger cues.

In our study, we demonstrate that ABCD1-deficient X-ALD macrophages, highly accumulating VLCFA lipid species, have increased expression of genes linked to JNK signalling, such as *c-FOS*, interacting protein 2 (*TRAF3IP2*), *TNF*, *IL1B* and *CXCL8* under steady-state conditions when compared to healthy controls. The extracellular exposure of healthy macrophages to free VLCFAs also triggered JNK signalling in a CD36-dependent manner, with robust expression and release of chemokines including CXCL8. Thus, contrary to the common theory proposing that saturated VLCFAs promote pro-inflammatory responses by stimulating TLR signalling, our data provide evidence refuting that C26:0, the saturated VLCFA accumulating most prominently in X-ALD patients, directly activates TLRs but rather stimulates pro-inflammatory macrophage response through the JNK pathway. Of note, the JNK pathway has been proposed as an important player also in other neurological disorders such as multiple sclerosis (MS). In MS, upregulation of JNK activity has been observed in peripheral blood mononuclear cells of relapsing patients when compared to healthy controls [58]. In addition, increased JNK phosphorylation, indicative of JNK signalling, was found in the acute disease phase of experimental autoimmune encephalomyelitis rats, an animal model mimicking neuroinflammation in MS [59]. Given the importance of the JNK pathway in pathological conditions, pan-JNK inhibition has been proposed as a novel treatment strategy for diseases associated with neurodegeneration and neuroinflammation [60, 61] and, thus, might also be of advantage in the context of X-ALD.

The rapidly progressive myelin destruction in patients with cerebral X-ALD is associated with a disturbed BBB and infiltration of peripheral monocytes/monocyte-derived macrophages and, to a lower extent, T cells [62]. Therefore, important insight can be derived from our observations of stimulated chemokine expression in VLCFA-accumulating X-ALD macrophages and enhanced release of chemokines from healthy control macrophages exposed to C26:0. These findings indicate a specific role of saturated VLCFAs in stimulating chemotaxis and possibly BBB transmigration to sites of brain inflammation. Upon crossing the brain endothelium, monocytes differentiate to macrophages and accumulate in perivascular cuffs. To reach the inflamed sites of the brain parenchyma, macrophages release enzymes to remodel the extracellular matrix at the basal lamina of the BBB. Consistently, our data revealed not only increased expression of chemokines and chemokine receptors in X-ALD macrophages but also increased mRNA levels for genes encoding adhesion molecules modulating the interaction of peripheral immune cells with the BBB (*THBS1*; *integrin* *subunit* *alpha* *L*, *ITGAL*) or proteins initiating the plasminogen cascade resulting in matrix turnover and cell invasion such as CXCL7. Further, increased VLCFA levels in X-ALD macrophages altered the expression of molecules associated with extracellular matrix remodelling (*CXCL5*, *PLAUR* and *hyaluronidase* *1*, *HYAL1*) and formation of membrane protrusions, as well as the modulation of BBB integrity for immune cell infiltration (CXCL8). Hence, our results pinpoint a mechanism by which VLCFA accumulation modulates X-ALD cells for invasive migratory behaviour. Our observation that macrophages respond to C26:0 treatment by increasing podosome formation lends further support for this concept. Of note, a recent report described reduced basal cell migration speed in a human neutrophil cell line with diminished VLCFA levels due to a knockdown of ELOVL1, the enzyme involved in VLCFA synthesis [63], thus further highlighting the role of VLCFAs in migratory behaviour. Whether indeed the high VLCFA levels in X-ALD macrophages amplify their ability to infiltrate the brain parenchyma upon onset of neuroinflammation remains to be clarified. Among the upregulated genes, *CCR2* encodes the main chemokine receptor required for monocyte migration from the bone marrow to inflammatory sites and plays a key role in the damaging effects of neuroinflammation following traumatic brain injury [64, 65]. Increased levels of CCR2 and CCL7 are not only observed in X-ALD macrophages, but have also been found in inflammatory brain lesions of X-ALD patients [66]. As classical anti-inflammatory treatment paradigms including application of IFNβ were unable to stop the neuroinflammation in X-ALD patients [67], pharmacological inhibition of factors involved in trans-endothelial migration across the BBB such as CCR2 or CXCL8 might be of interest in the context of developing alternative treatment strategies for the inflammatory cerebral form of X-ALD.

Taken together, we here show that the cellular content of saturated VLCFAs is tightly linked to the activation state of human macrophages and that ABCD1-mediated peroxisomal degradation of VLCFAs is pivotal for efficiently resolving the pro-inflammatory response. Thus, with saturated VLCFAs being linked to neuroinflammation and neurodegeneration, our findings on how these fatty acids modulate macrophage activation complements the current knowledge of lipid metabolism as a driving force in inflammation.

## Supplementary Information

**Additional file 1: Figure S1.** Flow cytometric analysis of CD14+ cell purity. **Figure S2.** Interactomes integrating dysregulated inflammatory response genes and protein–protein interactions related to cell surface receptors, plasma membrane proteins or G-protein coupled receptors in monocyte-derived macrophages from X-ALD patients versus healthy controls. **Figure S3.** Viability staining to evaluate cytotoxicity of C26:0 treatment in human primary macrophages. **Figure S4.** Inhibition of CD36 by SSO co-treatment reduces activation of the JNK pathway in C26:0-exposed macrophages. **Figure S5.** Pro-inflammatory *IL1B*, *TNF* and *IL6* cytokine expression is not significantly stimulated by VLCFA C26:0 treatment. **Figure S6.** C26:0 treatment affects chemokine release by human primary macrophages. **Figure S7.** Podosome formation in human primary macrophages treated with C26:0. **Table S1.** Primers used for RT-qPCR analysis

## Data Availability

The raw data and count tables used in RNA-seq analysis are available through NCBI's GEO repository under accession GSE217140. The R-markdown file used for the analysis of RNA-seq data presented in this paper is available in the repository: https://github.com/JureFabjan/macrophage_plasticity. All other data are included in the manuscript or in the Additional file 1 and are available from the corresponding author upon request.

## Abbreviations

25-HC: 25-Hydroxycholesterol
ABCD1: ATP-binding cassette subfamily D member 1
ATP: Adenosine triphosphate
BBB: Blood–brain barrier
BSA: Bovine serum albumin
C16:0: Palmitic acid
C24:1: Nervonic acid
C26:0: Hexacosanoic acid
C26:1: 17-Hexacosenoic acid
CALD: Cerebral X-linked adrenoleukodystrophy
CCL: C–C motif chemokine ligand
CCR: C–C chemokine receptor
CD36i: CD36 inhibitor
CH25H: Cholesterol-25-hydroxylase
CXCL: C-X-C motif chemokine ligand
DTT: Dithiothreitol
EDTA: Ethylenediamine tetraacetic acid
eGFP: Enhanced green fluorescent protein
ELOVL: Elongation of very long-chain fatty acids
ESI–MS: Electrospray ionization mass spectrometry
EtOH: Ethanol
FACS: Flow cytometry
FAD: Flavin adenine dinucleotide
FCS: Foetal calf serum
HClO_4_: Perchloric acid
HPRT1: Hypoxanthine phosphoribosyltransferase 1
HRP: Horseradish peroxidase
HSC: Haematopoietic stem cell
IFNγ: Interferon gamma
IL: Interleukin
JNK: C-Jun N-terminal kinase
KCl: Potassium chloride
KOH: Potassium hydroxide
LCFA: Long-chain fatty acids
LPS: Lipopolysaccharide
LXR: Liver X receptor
MALP2: Macrophage-activating lipopeptide-2
M-CSF: Macrophage colony-stimulating factor
MgCl_2_: Magnesium chloride
MMP: Matrix metalloproteinase
Na_3_VO_4_: Sodium orthovanadate
NAD+: Nicotinamide adenine dinucleotide
NaF: Sodium fluoride
NFκB: Nuclear factor kappa B
PAM3CSK4: Pam3CysSerLys4; synthetic triacylated lipopeptide
PBS: Phosphate-buffered saline
PLAUR: Urokinase plasminogen activator surface receptor
PMSF: Phenylmethylsulfonyl fluoride
PUFA: Polyunsaturated fatty acids
RACK1: Receptor for activated C kinase 1
RIN: RNA integrity number
RIPA buffer: Radio-immunoprecipitation assay buffer
RT-qPCR: Reverse transcription-quantitative polymerase chain reaction
SDS–PAGE: Sodium dodecyl-sulfate polyacrylamide gel electrophoresis
SSO: Sulfosuccinimidyl oleate
TBS-T: Tris-buffered saline-Tween
TLR: Toll-like receptor
TNF: Tumour necrosis factor
VLCFA: Very long-chain fatty acids
X-ALD: X-linked adrenoleukodystrophy

## References

[1] Hsieh W-Y, Zhou QD, York AG, Williams KJ, Scumpia PO, Kronenberger EB (2020). Toll-like receptors induce signal-specific reprogramming of the macrophage lipidome. Cell Metab.

[2] Dennis EA, Deems RA, Harkewicz R, Quehenberger O, Brown HA, Milne SB (2010). A mouse macrophage lipidome. J Biol Chem.

[3] Oishi Y, Spann NJ, Link VM, Muse ED, Strid T, Edillor C (2017). SREBP1 contributes to resolution of pro-inflammatory TLR4 signaling by reprogramming fatty acid metabolism. Cell Metab.

[4] Kanoh H, Nitta T, Go S, Inamori K-I, Veillon L, Nihei W (2020). Homeostatic and pathogenic roles of GM3 ganglioside molecular species in TLR4 signaling in obesity. EMBO J.

[5] Turk BR, Theda C, Fatemi A, Moser AB. X-linked adrenoleukodystrophy: pathology, pathophysiology, diagnostic testing, newborn screening, and therapies. Int J Dev Neurosci. 2019;25:S0736-5748(19)30133-9. 10.1016/j.ijdevneu.2019.11.002 31778737

[6] Berger J, Forss-Petter S, Eichler FS (2014). Pathophysiology of X-linked adrenoleukodystrophy. Biochimie.

[7] Weinhofer I, Rommer P, Zierfuss B, Altmann P, Foiani M, Heslegrave A (2021). Neurofilament light chain as a potential biomarker for monitoring neurodegeneration in X-linked adrenoleukodystrophy. Nat Commun.

[8] Raymond GV, Aubourg P, Paker A, Escolar M, Fischer A, Blanche S (2019). Survival and functional outcomes in boys with cerebral adrenoleukodystrophy with and without hematopoietic stem cell transplantation. Biol Blood Marrow Transplant.

[9] Cartier N, Hacein-Bey-Abina S, Bartholomae CC, Veres G, Schmidt M, Kutschera I (2009). Hematopoietic stem cell gene therapy with a lentiviral vector in X-linked adrenoleukodystrophy. Science.

[10] Eichler F, Duncan C, Musolino PL, Orchard PJ, De Oliveira S, Thrasher AJ (2017). Hematopoietic stem-cell gene therapy for cerebral adrenoleukodystrophy. N Engl J Med.

[11] Weber FD, Wiesinger C, Forss-Petter S, Regelsberger G, Einwich A, Weber WH (2014). X-linked adrenoleukodystrophy: very long-chain fatty acid metabolism is severely impaired in monocytes but not in lymphocytes. Hum Mol Genet.

[12] Weinhofer I, Zierfuss B, Hametner S, Wagner M, Popitsch N, Machacek C (2018). Impaired plasticity of macrophages in X-linked adrenoleukodystrophy. Brain.

[13] Zuercher WJ, Buckholz RG, Campobasso N, Collins JL, Galardi CM, Gampe RT (2010). Discovery of tertiary sulfonamides as potent liver X receptor antagonists. J Med Chem.

[14] Radakovics K, Battin C, Leitner J, Geiselhart S, Paster W, Stockl J (2021). A highly sensitive cell-based TLR reporter platform for the specific detection of bacterial TLR ligands. Front Immunol.

[15] Battin C, Hennig A, Mayrhofer P, Kunert R, Zlabinger GJ, Steinberger P (2017). A human monocytic NF-kappaB fluorescent reporter cell line for detection of microbial contaminants in biological samples. PLoS ONE.

[16] Dobin A, Davis CA, Schlesinger F, Drenkow J, Zaleski C, Jha S (2013). STAR: ultrafast universal RNA-seq aligner. Bioinformatics.

[17] Love MI, Huber W, Anders S (2014). Moderated estimation of fold change and dispersion for RNA-seq data with DESeq2. Genome Biol.

[18] Chen EY, Tan CM, Kou Y, Duan Q, Wang Z, Meirelles GV (2013). Enrichr: interactive and collaborative HTML5 gene list enrichment analysis tool. BMC Bioinform.

[19] Kuleshov MV, Jones MR, Rouillard AD, Fernandez NF, Duan Q, Wang Z (2016). Enrichr: a comprehensive gene set enrichment analysis web server 2016 update. Nucleic Acids Res.

[20] Zhou G, Soufan O, Ewald J, Hancock REW, Basu N, Xia J (2019). NetworkAnalyst 3.0: a visual analytics platform for comprehensive gene expression profiling and meta-analysis. Nucleic Acids Res.

[21] Valianpour F, Selhorst JJ, van Lint LE, van Gennip AH, Wanders RJ, Kemp S (2003). Analysis of very long-chain fatty acids using electrospray ionization mass spectrometry. Mol Genet Metab.

[22] Kim HN, Kim YR, Ahn SM, Lee SK, Shin HK, Choi BT (2015). Protease activated receptor-1 antagonist ameliorates the clinical symptoms of experimental autoimmune encephalomyelitis via inhibiting breakdown of blood–brain barrier. J Neurochem.

[23] Izikson L, Klein RS, Charo IF, Weiner HL, Luster AD (2000). Resistance to experimental autoimmune encephalomyelitis in mice lacking the CC chemokine receptor (CCR2). J Exp Med.

[24] Séguin R, Biernacki K, Rotondo RL, Prat A, Antel JP (2003). Regulation and functional effects of monocyte migration across human brain-derived endothelial cells. J Neuropathol Exp Neurol.

[25] Alter A, Duddy M, Hebert S, Biernacki K, Prat A, Antel JP (2003). Determinants of human B cell migration across brain endothelial cells. J Immunol.

[26] Biernacki K, Prat A, Blain M, Antel JP (2004). Regulation of cellular and molecular trafficking across human brain endothelial cells by Th1- and Th2-polarized lymphocytes. J Neuropathol Exp Neurol.

[27] Wilkinson H, Leonard H, Chen D, Lawrence T, Robson M, Goossens P (2021). PAR-1 signaling on macrophages is required for effective in vivo delayed-type hypersensitivity responses. iScience.

[28] Ghorbani S, Yong VW (2021). The extracellular matrix as modifier of neuroinflammation and remyelination in multiple sclerosis. Brain.

[29] Yamauchi Y, Kuroki M, Imakiire T, Uno K, Abe H, Beppu R (2002). Opposite effects of thrombospondin-1 via CD36 and CD47 on homotypic aggregation of monocytic cells. Matrix Biol.

[30] Song Z, Lv S, Wu H, Qin L, Cao H, Zhang B (2020). Identification of foam cell biomarkers by microarray analysis. BMC Cardiovasc Disord.

[31] Haarmann A, Schuhmann MK, Silwedel C, Monoranu C-M, Stoll G, Buttmann M (2019). Human brain endothelial CXCR2 is inflammation-inducible and mediates CXCL5- and CXCL8-triggered paraendothelial barrier breakdown. Int J Mol Sci.

[32] Petreaca ML, Yao M, Liu Y, DeFea K, Martins-Green M (2007). Transactivation of vascular endothelial growth factor receptor-2 by interleukin-8 (IL-8/CXCL8) is required for IL-8/CXCL8-induced endothelial permeability. Mol Biol Cell.

[33] Chrast R, Saher G, Nave K-A, Verheijen MH (2011). Lipid metabolism in myelinating glial cells: lessons from human inherited disorders and mouse models. J Lipid Res.

[34] Igarashi M, Schaumburg HH, Powers J, Kishmoto Y, Kolodny E, Suzuki K (1976). Fatty acid abnormality in adrenoleukodystrophy. J Neurochem.

[35] Lancaster GI, Langley KG, Berglund NA, Kammoun HL, Reibe S, Estevez E (2018). Evidence that TLR4 is not a receptor for saturated fatty acids but mediates lipid-induced inflammation by reprogramming macrophage metabolism. Cell Metab.

[36] Hammouda MB, Ford AE, Liu Y, Zhang JY (2020). The JNK signaling pathway in inflammatory skin disorders and cancer. Cells.

[37] Holzer RG, Park E-J, Li N, Tran H, Chen M, Choi C (2011). Saturated fatty acids induce c-Src clustering within membrane subdomains, leading to JNK activation. Cell.

[38] Drover VA, Nguyen DV, Bastie CC, Darlington YF, Abumrad NA, Pessin JE (2008). CD36 mediates both cellular uptake of very long chain fatty acids and their intestinal absorption in mice. J Biol Chem.

[39] Kuda O, Pietka TA, Demianova Z, Kudova E, Cvacka J, Kopecky J (2013). Sulfo-N-succinimidyl oleate (SSO) inhibits fatty acid uptake and signaling for intracellular calcium via binding CD36 lysine 164: SSO also inhibits oxidized low density lipoprotein uptake by macrophages. J Biol Chem.

[40] Polonskaya YV, Shramko V, Morozov S, Chernyak E, Chernyavsky A, Ragino Y (2017). Balance of fatty acids and their correlations with parameters of lipid metabolism and markers of inflammation in men with coronary atherosclerosis. Bull Exp Biol Med.

[41] Ofman R, Dijkstra IM, van Roermund CW, Burger N, Turkenburg M, van Cruchten A (2010). The role of ELOVL1 in very long-chain fatty acid homeostasis and X-linked adrenoleukodystrophy. EMBO Mol Med.

[42] Weinhofer I, Zierfuss B, Hametner S, Wagner M, Popitsch N, MacHacek C (2018). Impaired plasticity of macrophages in X-linked adrenoleukodystrophy. Brain.

[43] Zierfuss B, Weinhofer I, Buda A, Popitsch N, Hess L, Moos V (2020). Targeting foam cell formation in inflammatory brain diseases by the histone modifier MS-275. Ann Clin Transl Neurol.

[44] Zierfuss B, Weinhofer I, Kühl J-S, Köhler W, Bley A, Zauner K, et al. Vorinostat in the acute neuroinflammatory form of X-linked adrenoleukodystrophy. Ann Clin Transl Neurol. 2020;7(5):639–52. 10.1002/acn3.51015 PMC7261758 32359032

[45] Regan T, Gill AC, Clohisey SM, Barnett MW, Pariante CM, Harrison NA (2018). Effects of anti-inflammatory drugs on the expression of tryptophan-metabolism genes by human macrophages. J Leukoc Biol.

[46] Castrillo A, Tontonoz P (2004). Nuclear receptors in macrophage biology: at the crossroads of lipid metabolism and inflammation. Annu Rev Cell Dev Biol.

[47] Joseph SB, Bradley MN, Castrillo A, Bruhn KW, Mak PA, Pei L (2004). LXR-dependent gene expression is important for macrophage survival and the innate immune response. Cell.

[48] Liu Y, Wei Z, Ma X, Yang X, Chen Y, Sun L (2018). 25-Hydroxycholesterol activates the expression of cholesterol 25-hydroxylase in an LXR-dependent mechanism. J Lipid Res.

[49] Batista-Gonzalez A, Vidal R, Criollo A, Carreno LJ (2019). New insights on the role of lipid metabolism in the metabolic reprogramming of macrophages. Front Immunol.

[50] Wiesinger C, Kunze M, Regelsberger G, Forss-Petter S, Berger J (2013). Impaired very long-chain acyl-CoA beta-oxidation in human X-linked adrenoleukodystrophy fibroblasts is a direct consequence of ABCD1 transporter dysfunction. J Biol Chem.

[51] Weinhofer I, Buda A, Kunze M, Palfi Z, Traunfellner M, Hesse S (2022). Peroxisomal very long-chain fatty acid transport is targeted by herpesviruses and the antiviral host response. Commun Biol.

[52] Wei X, Song H, Yin L, Rizzo MG, Sidhu R, Covey DF (2016). Fatty acid synthesis configures the plasma membrane for inflammation in diabetes. Nature.

[53] Miller YI, Navia-Pelaez JM, Corr M, Yaksh TL (2020). Lipid rafts in glial cells: role in neuroinflammation and pain processing: thematic review series: biology of lipid rafts. J Lipid Res.

[54] Sviridov D, Mukhamedova N, Miller YI (2020). Lipid rafts as a therapeutic target: thematic review series: biology of lipid rafts. J Lipid Res.

[55] Guns J, Vanherle S, Hendriks JJ, Bogie JF (2022). Protein lipidation by palmitate controls macrophage function. Cells.

[56] Pradhan AJ, Lu D, Parisi LR, Shen S, Berhane IA, Galster SL (2021). Protein acylation by saturated very long chain fatty acids and endocytosis are involved in necroptosis. Cell Chem Biol.

[57] Liang X, Nazarian A, Erdjument-Bromage H, Bornmann W, Tempst P, Resh MD (2001). Heterogeneous fatty acylation of Src family kinases with polyunsaturated fatty acids regulates raft localization and signal transduction. J Biol Chem.

[58] Ferrandi C, Richard F, Tavano P, Hauben E, Barbié V, Gotteland J-P (2011). Characterization of immune cell subsets during the active phase of multiple sclerosis reveals disease and c-Jun N-terminal kinase pathway biomarkers. Mult Scler J.

[59] Shin T, Ahn M, Jung K, Heo S, Kim D, Jee Y (2003). Activation of mitogen-activated protein kinases in experimental autoimmune encephalomyelitis. J Neuroimmunol.

[60] Cui J, Zhang M, Zhang Y-Q, Xu Z-H (2007). JNK pathway: diseases and therapeutic potential. Acta Pharmacol Sin.

[61] Bagnoud M, Briner M, Remlinger J, Meli I, Schuetz S, Pistor M (2020). c-Jun N-terminal kinase as a therapeutic target in experimental autoimmune encephalomyelitis. Cells.

[62] Powers JM, Liu Y, Moser AB, Moser HW (1992). The inflammatory myelinopathy of adreno-leukodystrophy: cells, effector molecules, and pathogenetic implications. J Neuropathol Exp Neurol.

[63] Haney MS, Bohlen CJ, Morgens DW, Ousey JA, Barkal AA, Tsui CK (2018). Identification of phagocytosis regulators using magnetic genome-wide CRISPR screens. Nat Genet.

[64] Hsieh CL, Niemi EC, Wang SH, Lee CC, Bingham D, Zhang J (2014). CCR2 deficiency impairs macrophage infiltration and improves cognitive function after traumatic brain injury. J Neurotrauma.

[65] Somebang K, Rudolph J, Imhof I, Li L, Niemi EC, Shigenaga J (2021). CCR2 deficiency alters activation of microglia subsets in traumatic brain injury. Cell Rep.

[66] Paintlia AS, Gilg AG, Khan M, Singh AK, Barbosa E, Singh I (2003). Correlation of very long chain fatty acid accumulation and inflammatory disease progression in childhood X-ALD: implications for potential therapies. Neurobiol Dis.

[67] Korenke G, Christen H-J, Kruse B, Hunneman D, Hanefeld F (1997). Progression of X-linked adrenoleukodystrophy under interferon-β therapy. J Inherit Metab Dis.

