# Supplementary material for: Saturated very long-chain fatty acids regulate macrophage plasticity and invasiveness
Source: J Neuroinflammation. 2022 Dec 17;19:305. doi: 10.1186/s12974-022-02664-y (PMC9759912; doi:10.1186/s12974-022-02664-y)
Supplement: Supplementary file 1 — Additional file 1: Figure S1. Flow cytometric analysis of CD14+ cell purity. Figure S2. Interactomes integrating dysregulated inflammatory response genes and protein–protein interactions related to cell surface receptors, plasma membrane proteins or G-protein coupled receptors in monocyte-derived macrophages from X-ALD patients versus healthy controls. Figure S3. Viability staining to evaluate cytotoxicity of C26:0 treatment in human primary macrophages. Figure S4. Inhibition of CD36 by SSO co-treatment reduces activation of the JNK pathway in C26:0-exposed macrophages. Figure S5. Pro-inflammatory IL1B, TNF and IL6 cytokine expression is not significantly stimulated by VLCFA C26:0 treatment. Figure S6. C26:0 treatment affects chemokine release by human primary macrophages. Figure S7. Podosome formation in human primary macrophages treated with C26:0. Table S1. Primers used for RT-qPCR analysis [file 12974_2022_2664_MOESM1_ESM.pdf]

ADDITIONAL ELECTRONIC MATERIAL

Zierfuss et al. "SATURATED VERY LONG-CHAIN FATTY ACIDS REGULATE MACROPHAGE PLASTICITY AND INVASIVENESS"

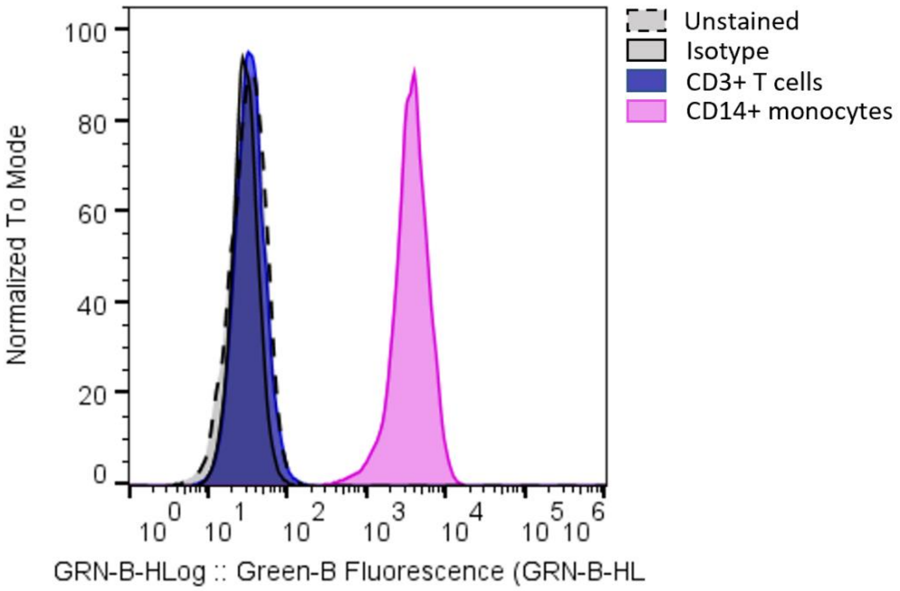

Supplementary Figure 1. Flow cytometric analysis of CD14+ cell purity. Freshly isolated CD14+ monocytes were stained with the fluorochrome-labelled antibodies CD14-FITC and CD3-FITC or the corresponding isotype control IgG2a-FITC. A representative flow cytometry analysis of purified monocytes derived from a leukoreduction system chamber from one healthy donor is shown. Monocytes were gated on forward versus side scatter, and the gated cell population was analysed for mean fluorescence intensity of FITC. The purity of the isolated CD14+ monocytes was ~99 %.

A

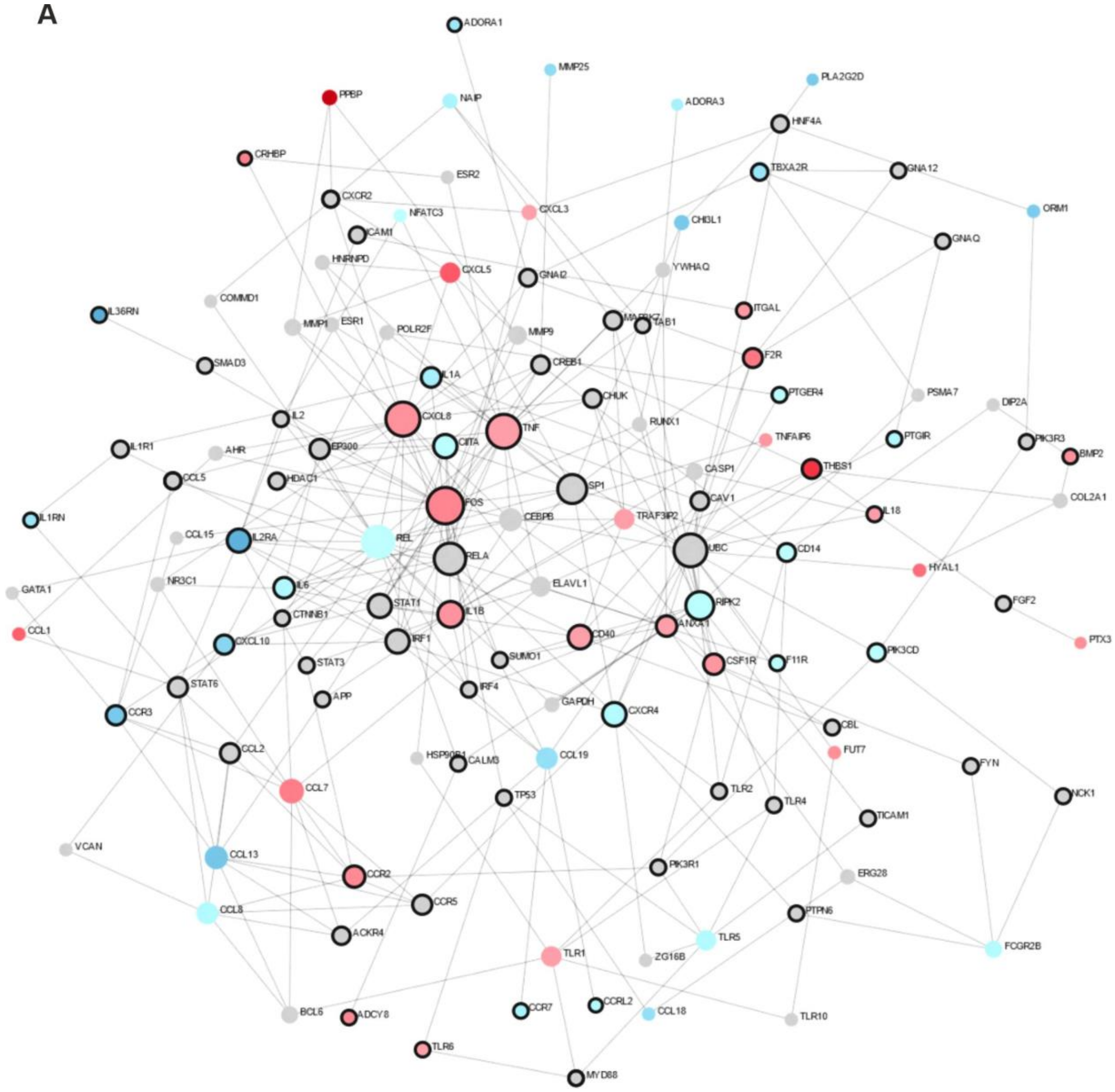
ADORA1
MMP25
PLA2G2D
PPBP
NAIP
ADORA3
HNF4A
CRHBP
ESR2
TBXA2R
GNA12
CXCR2
NFATC3
CXCL3
CHI3L1
ORM1
CAM1
GNAQ
HNRNPD
CXCL5
GNAI2
YWHAQ
COMMD1
IL36RN
MMP1
ESR1
POLR2F
MMP9
MA
TAB1
ITGAL
SMAD3
F2R
CREB1
IL1A
PTGER4
CHUK
PSMA7
DIP2A
IL2
CXCL8
TNF
RUNX1
IL1R1
AHR
EP300
CIITA
TNFAIP6
PTGIR
PIK3R3
BMP2
CCL5
HDAC1
CASP1
THBS1
COL2A1
CAV1
SP1
FOS
CEBPB
TRAF3IP2
IL18
IL1RN
CCL15
IL2RA
REL
UBC
CD14
HYAL1
GATA1
NR3C1
RELA
IL6
ELAVL1
RIPK2
FGF2
CCL1
CTNNB1
STAT1
IL1B
ANXA1
CD40
PTX3
CXCL10
IRF1
SUMO1
PIK3CD
STAT3
CSF1R
F11R
STAT6
APP
IRF4
CCR3
GAPDH
CXCR4
CBL
CCL2
FUT7
HSP90B
CALM3
CCL19
FYN
CCL7
TP53
TLR2
TLR4
NCK1
TICAM1
VCAN
CCL13
CCR2
PIK3R1
ERG28
CCR5
PTPN6
CCL8
ACKR4
TLR5
FCGR2B
TLR1
ZG16B
BCL6
ADCY8
CCR7
CCRL2
CCL18
TLR10
TLR6
MYD88

B

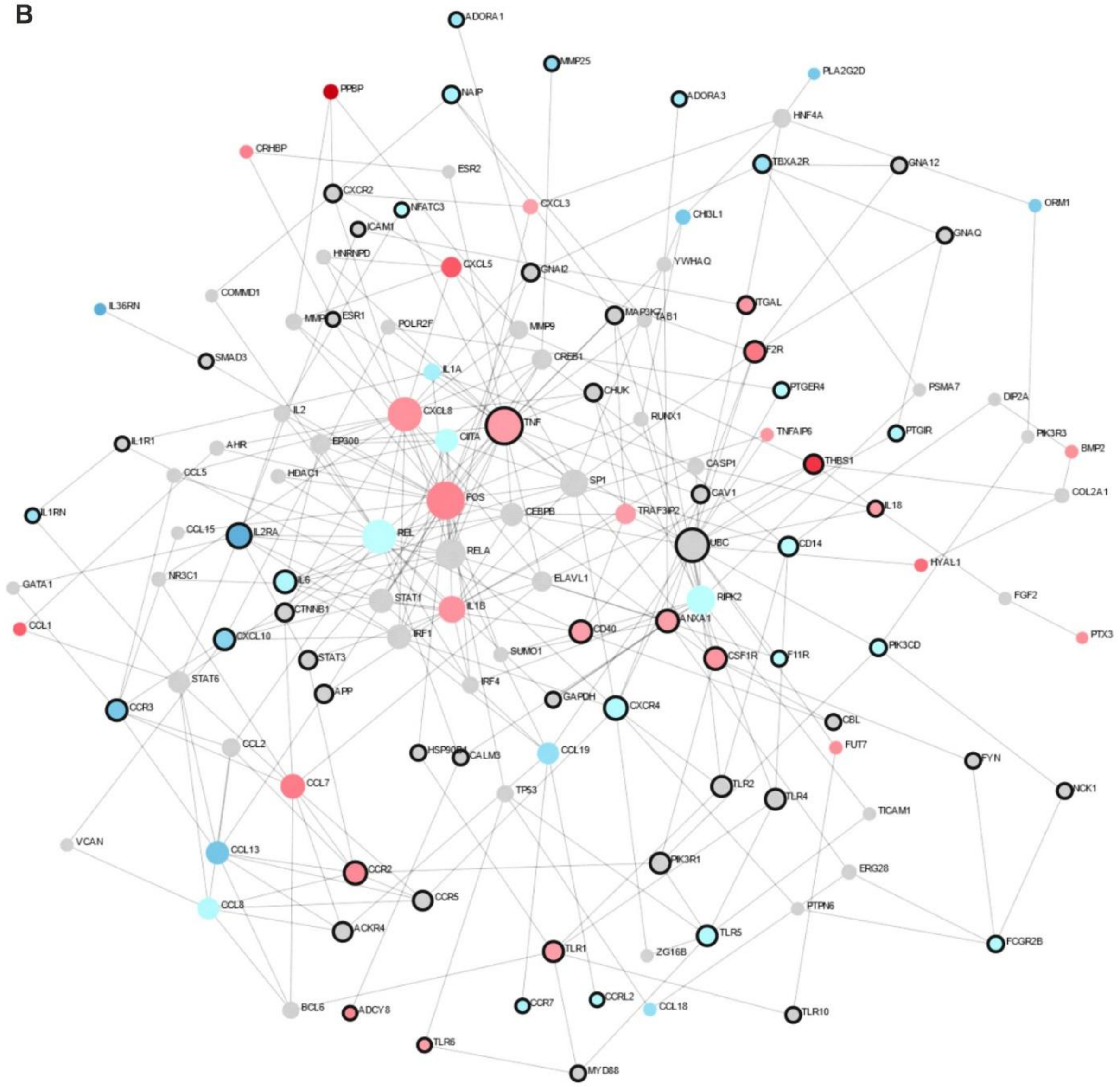
ADORA1
MMP25
PPBP
NAIP
PLA2G2D
ADORA3
HNF4A
CRHBP
ESR2
TBXA2R
GNA12
CXCR2
NFATC3
CXCL3
CHI3L1
ORM1
ICAM1
GNAQ
HNRNPD
CXCL5
GNAI2
YWHAQ
COMMD1
IL36RN
ITGAL
ESR1
POLR2F
MMP9
MAP3K7
TAB1
F2R
SMAD3
CREB1
IL1A
CHUK
PTGER4
PSMA7
IL2
CXCL8
TNF
RUNX1
DIP2A
IL1R1
AHR
EP300
CIITA
TNFAIP6
PTGIR
PIK3R3
BMP2
CCL5
HDAC1
CASP1
THBS1
SP1
CAV1
COL2A1
IL1RN
FOS
CEBPB
TRAF3IP2
IL18
CCL15
IL2RA
REL
UBC
CD14
RELA
HYAL1
GATA1
NR3C1
IL6
ELAVL1
RIPK2
FGF2
STAT1
CTNNB1
IL1B
ANXA1
CCL1
CD40
PTX3
CXCL10
IRF1
PIK3CD
SUMO1
CSF1R
F11R
STAT3
STAT6
APP
IRF4
GAPDH
CXCR4
CCR3
CBL
FUT7
CCL2
CALM3
CCL19
FYN
CCL7
TP53
TLR2
TLR4
NCK1
TICAM1
VCAN
CCL13
PIK3R1
ERG28
CCR2
CCR5
CCL8
PTPN6
ACKR4
TLR5
FCGR2B
TLR1
ZG16B
BCL6
ADCY8
CCR7
CCRL2
CCL18
TLR10
TLR6
MYD88

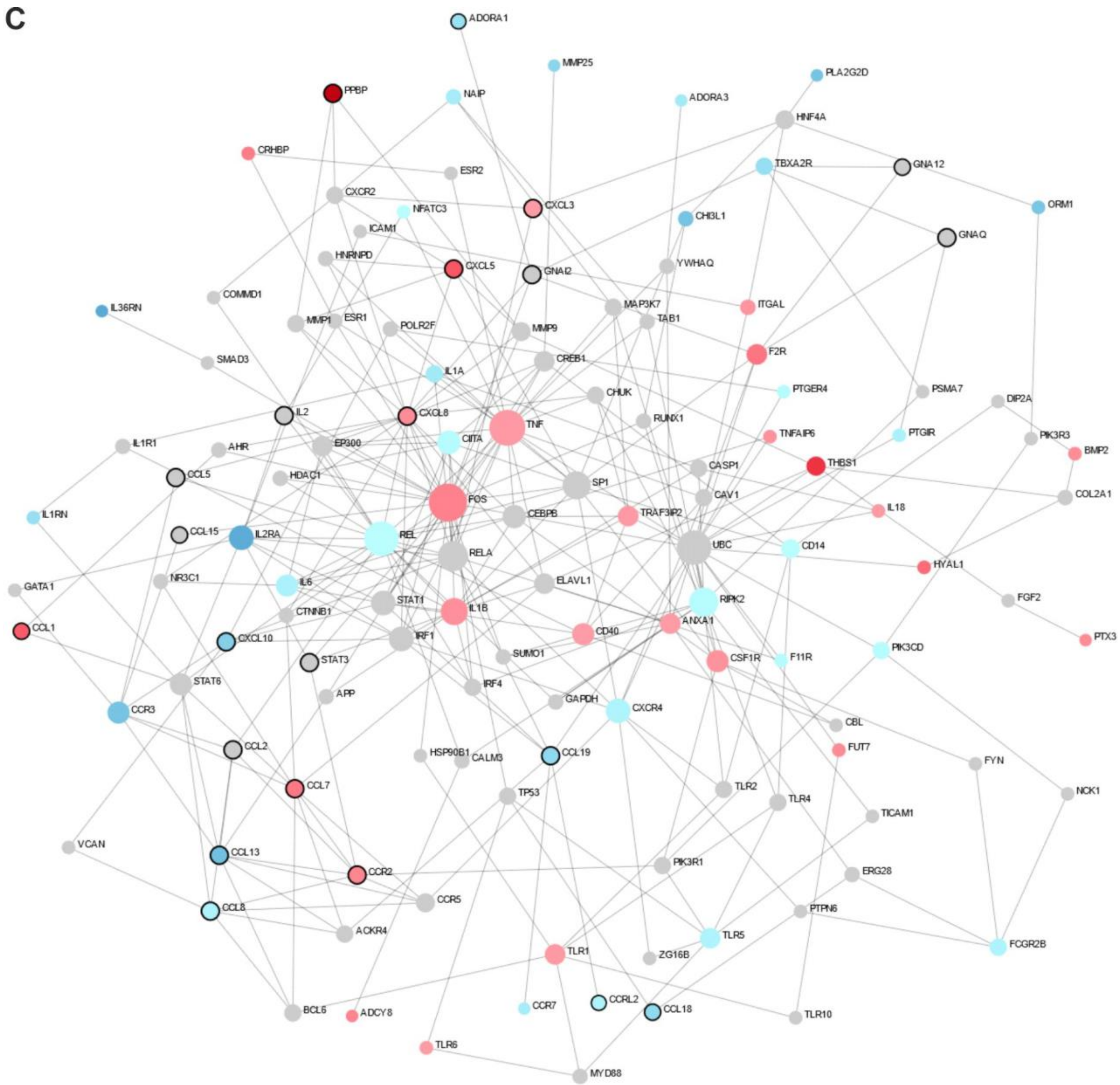

Supplementary Figure 2. Interactomes integrating dysregulated inflammatory response genes and protein-protein interactions related to cell surface receptors, plasma membrane proteins or G-protein coupled receptors in monocyte-derived macrophages from X-ALD patients versus healthy controls. Nodes represent genes in the inflammatory response term of Gene Ontology (GO:0006954), while edges show protein-protein interactions between gene products. Black encircled nodes depict subsets related to: **(A)** the cell surface receptor signalling pathway GO:BP, **(B)** the plasma membrane GO:CC and **(C)** the G-protein coupled receptor binding GO:MF. Genes that RNA-Seq analysis revealed as significantly dysregulated between monocyte-derived macrophages from X-ALD patients and healthy controls (*n*=9 each) have coloured nodes. Specifically, the red node colour depicts log2-fold upregulated genes, blue log2-fold downregulated genes and grey non-differentially expressed genes. The frequency of protein-protein interaction is reflected by the size of the node.

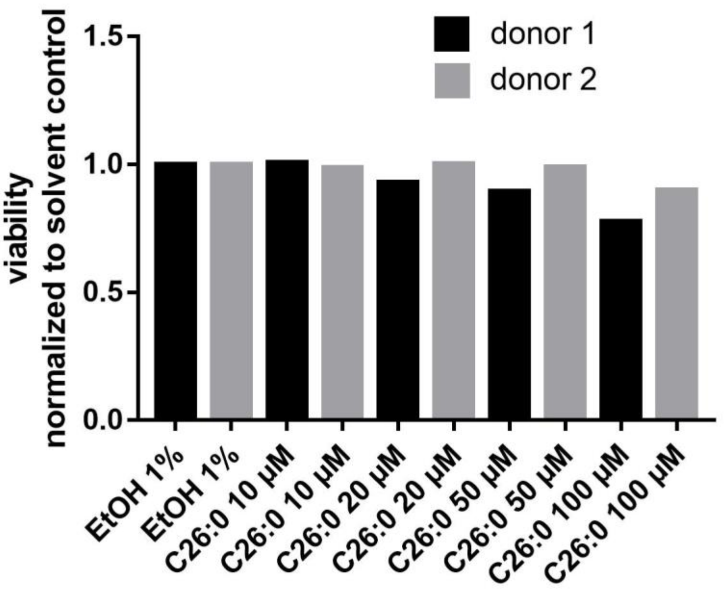

Supplementary Figure 3. Viability staining to evaluate cytotoxicity of C26:0 treatment in human primary macrophages. Monocyte-derived macrophages from two healthy donors were incubated with the indicated concentrations of C26:0 or the solvent (ethanol, EtOH) for 24 hrs before the viability of macrophages was assessed using Calcein Red-AM staining.

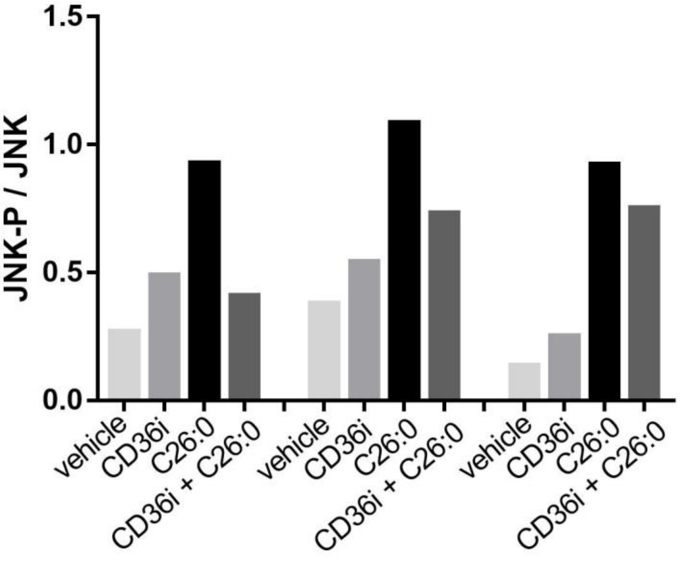

Supplementary Figure 4. Inhibition of CD36 by SSO co-treatment reduces activation of the JNK pathway in C26:0-exposed macrophages. The absolute ratios of the signal intensities for phosphorylated and total JNK in Fig. 2H derived from macrophages of three healthy donors incubated with either C26:0 (100 µM), the CD36 inhibitor (CD36i) sulfosuccinimidyl oleate (100 µM) or both compounds for 24 hrs prior to immunoblotting for detection of phosphorylated and total JNK1/JNK2.

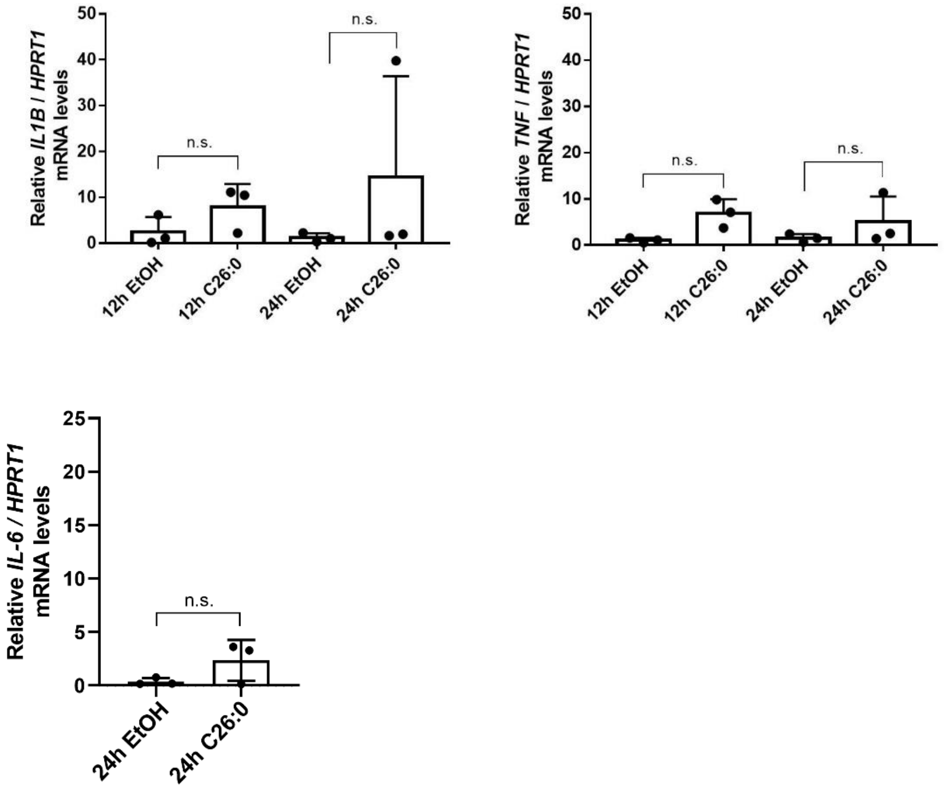

Supplementary Figure 5. Pro-inflammatory *IL1B, TNF* and *IL6* cytokine expression is not significantly stimulated by VLCFA C26:0 treatment. Human macrophages derived from healthy control donors (*n*=3) were treated with 100 µM C16:0 or 100 µM C26:0 or EtOH (vehicle) as solvent control for 12 and 24 hrs before RT-qPCR was carried out to measure mRNA levels for *IL1B*, *TNF* or *IL6* and, for normalization, *HPRT1*. Student´s paired *t*-test was was used for statistical analysis. Ns = not significant.

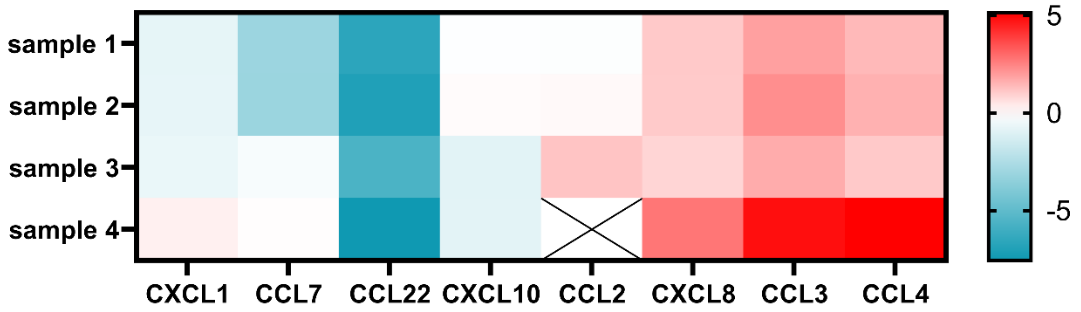

Supplementary Figure 6. C26:0 treatment affects chemokine release by human primary macrophages. Healthy control macrophages (*n*=4) were treated with C26:0 (100 µM) or the solvent EtOH for 24 hrs. Supernatants were analysed using Luminex ELISA bead assay for the chemokines CXCL1, CCL7, CCL22, CXCL10, CCL2, CXCL8, CCL3, and CCL4 (shown) and CCL11, CX3CL1, IL-12p40 and IL-15 (not shown), for which the levels were below the detection limit. The heat map indicates log fold changes to solvent-treated samples.

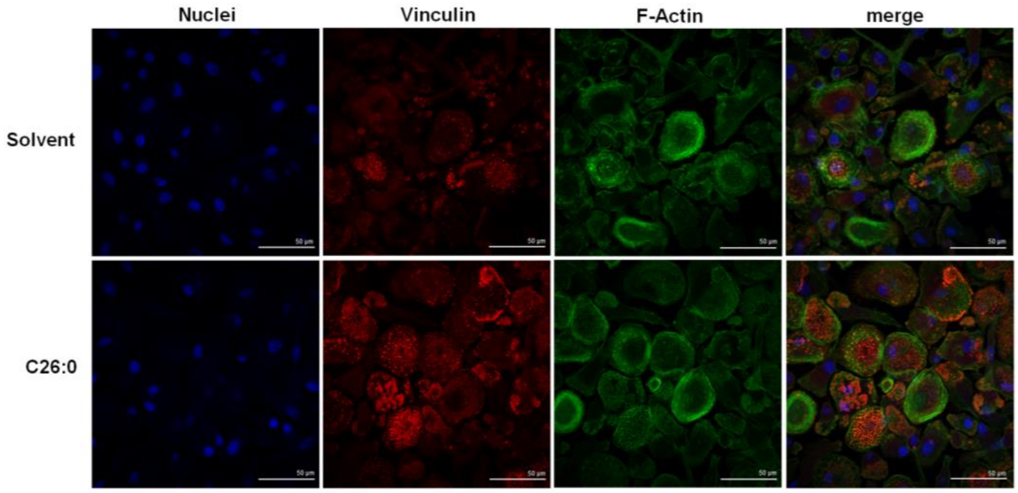

Supplementary Figure 7. Podosome formation in human primary macrophages treated with C26:0. Confocal laser scanning micrographs of human primary macrophages derived from a healthy donor. Cells were differentiated on glass slides for 7 days and treated with either C26:0 (100 µM) or the solvent EtOH for 24 hrs before fluorescence microscopy was carried out. Podosomes were visualized using an antibody directed against the actin binding protein vinculin (red colour) and AlexaFluor^{488}-labeled phalloidin staining F-actin (green colour). Nuclei were stained with DAPI.

Supplementary Table 1. Primers used for RT-qPCR analysis

Gene	Accession number	Product length [bp]	Sequence
ABCD1	NM_000033	169	F 5´- gagaacatccccatcgtc -3´<br>R 5´- tgtagagcacaccaccgta -3´
ABCD3	M81182	98	F 5´- cggctcatcacaaacagtga -3´<br>R 5´- aggtgttccaccagttttcg -3´
ACOX1	NM_004035	126	F 5´- cctggtgggcttggaaaga -3´<br>R 5´- caaaggcttatgggtcccga -3´
CCL2	NM_002982	100	F 5´- agctgtgatcttcaagaccattg -3´<br>R 5´- ttgggtttgcttgtccaggt -3´
CCL3	NM_002983	206	F 5´- agcccggtgtcatcttccta -3´<br>R 5´- agaggtagctgtggaggtca -3´
CCL4	NM_002984	133	F 5´- tgtggtattccaaaccaaaaga -3´<br>R 5´- caggtgaccttccctgaaga -3´
CH25H	NM_003956	263	F 5´- accacatcctgttctgcctg -3´<br>R 5´- ctccacggaaagccagatgt -3´
CXCL8	NM_000584	200	F 5´- ctctgtgtgaaggtgcagttt -3´<br>R 5´- ccagttttccttggggtcca -3´
ELOVL1	NM_022821	279	F 5´- attgagctgatggacacagtgat -3´<br>R 5´- gaccaggacaaactggatcagc -3´
ELOVL7	NM_024930	77	F 5´- ggccagcctaccagaagtatttg -3´<br>R 5´- ggcgacaataacaaactggacaag -3´
FADS2	NM_004265	202	F 5´- tcctcaacttcatcaggttcct -3´<br>R 5´- gggaagaggtggtgctcaat -3´
FASN	NM_004104	141	F 5´- gcaagctgaaggacctgtct -3´<br>R 5´- aatctgggttgatgcctccg -3´
HPRT1	NM_000194	220	F 5´- ccctggcgtcgtgattagt -3´<br>R 5´- caggtcagcaaagaatttatagcc -3´
HSD17B4	NM_002153	207	F 5´- atgctgcaggacagaggact -3´<br>R 5´- ccctcctcccatgctgct -3´
IL12B	NM_002187	264	F 5´- tcatcagggacatcatcaaacct -3´<br>R 5´- tatagtagcggtcctgggcc -3´
IL1B	NM_000576	213	F 5´- gcttggtgatgtctggtcca -3´<br>R 5´- tgggatctacactctccagct -3´
IL6	NM_000600	164	F 5´- catcctcgacggcatctcag -3´<br>R 5´- tcaccaggcaagtctcctca -3´
MMP9	NM_004994	218	F 5´- ggcgtcgtggttccaact -3´<br>R 5´- ggtttcccatcagcattgcc -3´
MMP14	NM_004995	183	F 5´- gagaagcaggccgacatcat -3´<br>R 5´- gtcatttccattcagatcctcattcc -3´
NR1H3 / LXRα	NM_001130101	261	F 5´- cagggctgcaagtggaattca -3´<br>R 5´- tccggaggctcaccagtttc -3´
PLAUR	NM_002659	185	F 5´- cgcttgtgggaagaaggaga -3´<br>R 5´- cggcttcgggaataggtgac -3´
SCD1	NM_005063	170	F 5´- ttctacacctggctttgggg -3´<br>R 5´- gcccattcatagacatcattctgg -3´
TNF	NM_000594	250	F 5´- cccaggcagtcagatcatct -3´<br>R 5´- ctgatggtgtgggtgaggag -3´
